# Our unknown neighbor: A new species of rain frog of the genus *Pristimantis* (Amphibia: Anura: Strabomantidae) from the city of Loja, southern Ecuador

**DOI:** 10.1371/journal.pone.0258454

**Published:** 2021-10-27

**Authors:** Paul Székely, Diana Székely, Leonardo Ordóñez-Delgado, Diego Armijos-Ojeda, Judit Vörös

**Affiliations:** 1 Museo de Zoología, Universidad Técnica Particular de Loja, Loja, Ecuador; 2 Departamento de Ciencias Biológicas y Agropecuarias, Laboratorio de Ecología Tropical y Servicios Ecosistémicos (EcoSs-Lab), Facultad de Ciencias Exactas y Naturales, Universidad Técnica Particular de Loja, Loja, Ecuador; 3 Programa de Doctorado en Conservación de Recursos Naturales, Escuela Internacional de Doctorado, Universidad Rey Juan Carlos, Madrid, Spain; 4 Department of Zoology, Hungarian Natural History Museum, Budapest, Hungary; Guangxi University, CHINA

## Abstract

We describe a new species of rain frog of the genus *Pristimantis* from the city of Loja, Southern Ecuador, based on an integrative taxonomy approach, combining molecular, morphological and bioacoustics data. *Pristimantis lojanus* sp. nov. is a medium sized species of the phylogenetically strongly supported *P*. *phoxocephalus* group, and its sister species is *P*. *torresi*. The new species can be easily distinguished from its closest congeners and morphologically similar species (that also have acuminate snout with a fleshy keel) by its characteristic advertisement call and morphological features (dorsum finely tuberculate with scattered larger tubercles, flanks without longitudinal lateral folds, no markings in axilla, groin or on concealed limb surfaces, and bronze iris). Additionally, we describe the advertisement call of its sister species, *P*. *torresi*. Finally, we detail the current situation of the amphibian species present in the city of Loja and its surroundings.

## Introduction

Ecuador is one of the most biodiverse countries in the World [1, 2], having the highest density of amphibian species—number of species per area unit [3], as well as a remarkable proportion of endemic species [4]. Despite its small size, Ecuador harbors several types of ecosystems in the Tropical Andes region, which have been recognized as hotspots of amphibian diversity [3]. Their distinctiveness is still far from completely known, numerous species being described during the recent years [5–8]. The city of Loja, the capital of Loja Province, is one of the largest cities from Southern Ecuador (with more than 250,000 inhabitants), and has two areas of high diversity in its vicinity: Parque Nacional Podocarpus and Abra de Zamora [7, 9].

Loja itself has a fascinating history of amphibian records reported from the city and its surroundings. In 1932, the British zoologist Hampton Wildman Parker [10] described the frog *Eleutherodactylus carrion*, with specimens collected by the Ecuadorian naturalist Clodoveo Carrión Mora from the city. However, in 1969, John D. Lynch [11] would synonymize it with *Eleutherodactylus lymani*, currently *Pristimantis lymani* [12]. Parker, in the same paper from 1932 [10], described, with specimens collected by Carrión from the city, a new subspecies of marsupial frogs, *Gastrotheca marsupiata lojana*, which in turn would be elevated to species status by William E. Duellman in 1974 [13] as *Gastrotheca lojana*. As an interesting note, the same Parker wrote in 1934 [14] and 1938 [15] about specimens of *Atelopus ignescens* collected by Carrión from the city of Loja, although the identity of these specimens is yet to be determined, as the probable distribution of this Critically Endangered species is only in Northern Ecuador [16].

In 1971, Edwards [17] described a poison frog, the Loja Rocket Frog (*Colostethus elachyhistus*, currently *Hyloxalus elachyhistus*), with specimens collected by Lynch in 1968, and in 2003 Pramuk and Kadivar [18] described a new species of toad, *Bufo amabilis* (currently *Rhinella amabilis* and probably the same species mentioned by Parker as *Bufo spinulosus* in 1934 [14]), with specimens collected by Lynch from Loja in 1968. And, in 2019, Carvajal-Endara et al. [19] described another species of marsupial frogs, *Gastrotheca elicioi*, from the city and its surroundings.

In 1979, Lynch [20] described a new species, *Pristimantis phoxocephalus* (from Pilaló, Cotopaxi Province), listing among the examined specimens also animals collected from Loja. This species was long considered a single highly polymorphic species (e.g. [21, 22]), with an extensive distribution ranging from Peru to Northern Western Ecuador. However, in 2019 Páez & Ron [23] showed that many populations represent in fact different, undescribed species. Based on integrative evidence, using molecular, morphological and bioacoustic data, we here describe a new species of the *P*. *phoxocephalus* group (sensu [23]) from the city of Loja.

## Materials and methods

### Ethics statement

This study was carried out in strict accordance with the guidelines for use of live amphibians and reptiles in field research compiled by the American Society of Ichthyologists and Herpetologists, the Herpetologists’ League and the Society for the Study of Amphibians and Reptiles. Research permits were issued by the Ecuadorian Ministry of Environment (MAE-DNB-CM-2015-0016, MAAE-ARSFC-2020-0727 and MAATE–DZ7L-GBVS-046-2021). This study was evaluated and approved by the Ethics Committee of Universidad Técnica Particular de Loja (UTPL-CBEA-2016-001).

### Specimen collection and study site

Fieldwork was carried out irregularly between October 2012 and May 2021 in and around the city of Loja (Loja province, southern Ecuador; 4.0007° S, 79.2045° W, datum WGS84; 2070 m above sea level) and in several neighboring areas: San Lucas and its vicinities (about 35 km to the north; 3.7101° S, 79.2545° W, 2748 m), Bosque Servio Aguirre Villamagua (about 20 km to the north; 3.8147° S, 79.2948° W, 2800 m), Cerro Sacama (about 7 km to the north; 3.8992° S, 79.2577° W, 2583 m), Abra de Zamora (about 8 km to the east; 3.9873° S, 79.1518° W, 2406 m), Cajanuma, on the road to Parque Nacional Podocarpus entrance (about 8 km to the south; 4.1123° S, 79.1820° W, 2660 m), Cristal (about 10 km to the south; 4.1248° S, 79.1928° W, 2043 m), and San Antonio de Paycapamba (about 12 km to the south; 4.1348° S, 79.2484° W, 2675 m). In the sampled area we made intensive visual encounter surveys and auditory surveys both during the day and during the night (12h00–02h00).

Collected specimens were photographed alive, after which they were euthanized using 20% benzocaine, fixed in 10% formalin, and stored in 70% ethanol and the tissue samples for genetic analyses were preserved in 96% ethanol [7]. Examined specimens (listed in the type-series and S1 Appendix) are housed in Museo de Zoología, Universidad Técnica Particular de Loja, Loja, Ecuador (MUTPL).

### Morphology

The description of qualitative and quantitative morphological characters, as well as the format of the description largely follows Duellman & Lehr [22]. Sex was determined by the presence of vocal slits, nuptial pads and/or by gonadal inspection. Color data in life were based on field notes and digital photos. The specimens were weighted (body mass: BM) before euthanasia using a My Weigh Triton T3 portable scale with 0.01 g precision. Measurements were taken under a stereo microscope, with a Vernier caliper, and rounded to the nearest 0.1 mm. Specimens were measured for the following morphometric variables: (1) snout-vent length (SVL), distance from the tip of snout to posterior margin of vent; (2) head width (HW), widest portion of the head, measured at level of jaw articulation; (3) head length (HL), distance from the tip of snout to posterior angle of jaw articulation; (4) interorbital distance (IOD), minimum distance between the inner margins of the orbits; (5) internarial distance (IND), distance between the inner edges of the narial openings; (6) upper eyelid width (EW), the perpendicular distance to the outer edge of the eyelid; (7) eye diameter (ED), distance between anterior and posterior borders of eye; (8) eye-nostril distance (EN), distance from posterior margin of nostril to anterior margin of eye; (9) tympanum diameter (TD), horizontal distance between peripheral borders of tympanic annulus; (10) femur length (FL), length of femur from vent to knee; (11) tibia length (TL), length of flexed leg from knee to heel; (12) foot length (FoL), distance from proximal margin of inner metatarsal tubercle to tip of Toe IV; (13) hand length (HaL), distance from proximal edge of palmar tubercle to the tip of Finger III [7]. Measurements are given as mean ± SD.

### DNA extraction, amplification and sequencing

DNA extraction was performed directly from 96% ethanol-preserved liver tissue, using the Extract-N-Amp™ Tissue PCR Kit (Sigma-Aldrich, Merck KGaA, Darmstadt, Germany), followed by PCR reactions under the manufacturer’s protocol [7]. Genomic extraction, amplification, and sequencing are as described in Székely et al. [7]. The newly generated DNA sequences (*12S*, *16S* and *RAG-1*) were deposited in GenBank (S1 Table).

### DNA sequence analyses

Molecular data were analyzed using sequences of two mitochondrial genes (*12S* and *16S* rRNA) and one nuclear gene (*RAG-1*, recombination-activating gene 1) from 52 individuals of 30 species from Ecuador (S1 Table), representing all the currently confirmed species of the *Huicundomantis* subgenus of *Pristimantis* [23]. We used the GenBank-available sequences for the *Huicundomantis* [7, 23], and 15 new sequences (of 3 species) generated in the current study (S1 Table). As outgroups we used *Pristimantis unistrigatus*, *P*. *ceuthospilus*, *P*. *imitatrix*, *P*. *diadematus*, *P*. *rhodoplichus*, *P*. *melanogaster*, *P*. *wiensi*, *P*. *simonsii*, *P*. *orestes*, *P*. *colodactylus*, and *P*. *orcesi*. The trees were rooted with *P*. *galdi*.

The sequences were edited, assembled, and aligned (MAFFT algorithm [24]) using the program Geneious Prime (Biomatters Ltd.). The edited alignments of *12S*, *16S* and *RAG-1* sequences were manually inspected and concatenated into a single matrix, which was then used for the phylogenetic analyses [7]. The phylogenetic analyses were based on a 2339 bp dataset (909 bp *12S*, 878 bp *16S* and 612 bp *RAG-1*). The aligned matrix is available at https://doi.org/10.5281/zenodo.5500445.

Molecular phylogenetic relationships were inferred using Maximum Likelihood (ML) and Bayesian Inference (BI). We used PartitionFinder v. 2.1.1 [25] to select the best-fitting models of sequence evolution and the best partition scheme with the AICc (for ML) and BIC (for BI) models of selection. ML analyses were conducted in GARLI v. 2.1 [26], performing four independent searches (two with the “streefname” set to random and two set to stepwise), with 250 replicates each, and with the “genthreshfortopoterm” set to 100,000 [7]. Node support was assessed with non-parametric bootstrapping [27] with 1,000 pseudoreplicates. The 50% majority rule consensus for the bootstrap trees was obtained with Geneious Prime (Biomatters Ltd.). BI analyses were conducted with MrBayes 3.2.6 [28], the Markov chain Monte Carlo runs being performed twice, independently, for 60 million generations, with a sampling frequency of 1000. Convergence of the runs was assessed from the average split frequency of standard deviations (*p* < 0.001) and by checking the potential scale reduction factors (PSRF ~ 1.0) for all model parameters. The first 25% of the trees were discarded as burn-in and the remaining ones were used to generate a 50% majority rule consensus tree, as well as to estimate the Bayesian posterior probabilities [7]. Throughout the text, we considered that a node has “strong support” when its bootstrap value was > 70 and its Bayesian posterior probability was > 0.95, “moderate support” for 50–70 and 0.90–0.95 and “weak support” or non-resolved for lower values of 50 or 0.90, respectively [7]. Uncorrected *p*-genetic distances for gene 16S (S2 Table) were estimated with software MEGA6 [29].

### Call recordings and analysis

The calls were recorded in the field using an Olympus LS-11 Linear PCM Recorder, a Tascam DR-100 MKIII Recorder and a RØDE NTG2 condenser shotgun microphone at 44.1 kHz sampling frequency and 16-bit resolution, in WAV file format. Air temperature and humidity were measured with a data logger (Lascar Electronics, model EL-USB-2-LCD, accuracy: ± 0.5°C; ± 5%). All analyzed call recordings are deposited in original form, full length at Fonoteca UTPL (record IDs are provided in S3 Table). Acoustic analysis was conducted using Raven Pro 1.6 (Center for Conservation Bioacoustics 2019). We measured the temporal parameters from the oscillograms and the spectral parameters from spectrograms obtained with the Hanning window function, DFT: 512 samples, 3 dB filter bandwidth: 124 Hz, and a 50% overlap [7].

The terminology and procedures for measuring call parameters follow [30–32], with a call-centered approach to distinguish between a call and a note (sensu [32]). The following temporal and spectral parameters were measured and analyzed: (1) *call duration*: time from the beginning to the end of a call; (2) *inter-call interval*: the interval between two consecutive calls, measured from the end of one call to the beginning of the consecutive call; (3) *call rate*: number of calls per minute, measured as the time between the beginning of the first call and the beginning of the last call; (4) *note duration*: the duration of a single note within a call, measured from beginning to the end of the note; (5) *inter-note interval*: the interval between two consecutive notes within the same call, measured from the end of one note to the beginning of the consecutive note; (6) *note rate*: number of notes per second, measured as the time between the beginning of the first note and the beginning of the last note; (7) *dominant frequency*: the frequency containing the highest sound energy, measured along the entire call; and (8) *the 90% bandwidth*, reported as *frequency 5%* and *frequency 95%*, or the minimum and maximum frequencies, excluding the 5% below and above the total energy in the selected call [7].

### Nomenclatural acts

The electronic edition of this article conforms to the requirements of the amended International Code of Zoological Nomenclature, and hence the new names contained herein are available under that Code from the electronic edition of this article. This published work and the nomenclatural acts it contains have been registered in ZooBank, the online registration system for the ICZN. The ZooBank LSIDs (Life Science Identifiers) can be resolved and the associated information viewed through any standard web browser by appending the LSID to the prefix "http://zoobank.org/". The LSID for this publication is: urn:lsid:zoobank.org:pub:4C8C6141-DC8A-4A0E-818E-B87B0F655278. The electronic edition of this work was published in a journal with an ISSN, and has been archived and is available from the following digital repositories: PubMed Central and LOCKSS.

## Results

### Phylogeny

**Fig 1:**
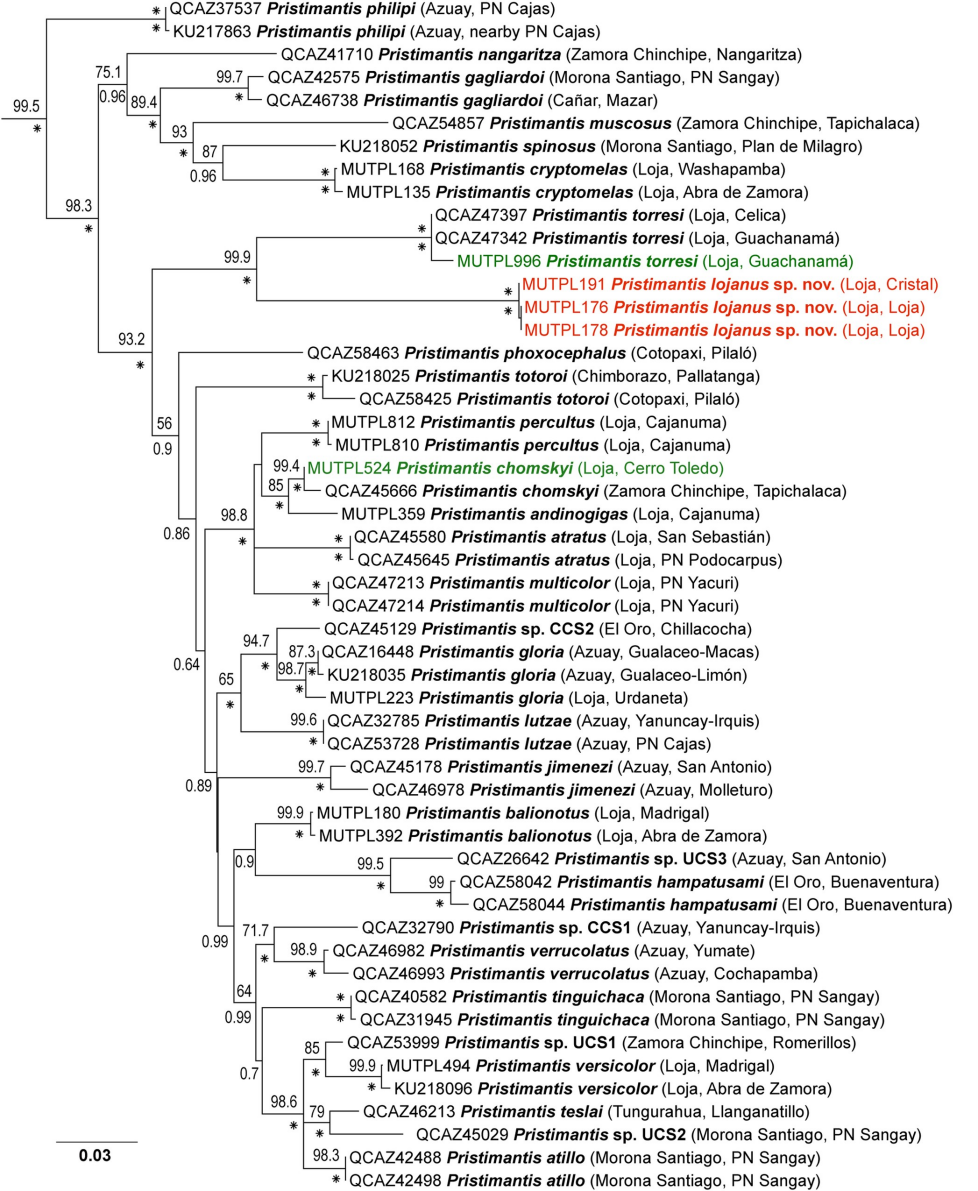
Maximum likelihood phylogram of the *Huicundomantis* subgenus of *Pristimantis* based on 2339 base pairs of concatenated DNA from *12S*, *16S*, and *RAG-1* gene fragments. Bootstrap values (%) and Bayesian posterior probabilities (decimal) are shown except when they are below 50 (bootstrap) or 0.5 (posterior probability); asterisks indicate support values of 100% or 1 (posterior probabilities). Outgroup is not shown; the tree was rooted with *Pristimantis galdi*. With red is marked the new species and with green newly generated sequences for the present study. The collection number, species name, province and short locality name of the samples are shown next to each terminal; all samples are from Ecuador (associated data listed in S1 Table). Abbreviations: CCS = confirmed candidate species, UCS = unconfirmed candidate species (see [23]).

PartitionFinder under AICc (for ML) identified four partition schemes as the best strategy (best model in parentheses): *12S* and *16S* (GTR+I+G), *RAG-1* 1^st^ position (K81UF+G), *RAG-1* 2^nd^ position (TVM+I+G), and *RAG-1* 3^rd^ position (TIM+I). Under BIC (for BI) PartitionFinder identified three partitions: *12S* and *16S* (GTR+I+G), *RAG-1* 1^st^ position (K80+G), and *RAG-1* 2^nd^ and 3^rd^ positions (F81+I+G). The phylogenetic trees constructed by Bayesian inference and Maximum likelihood showed almost the same topology (with some minor differences in the position of some of the unresolved branches), but with overall stronger support in the case of the Bayesian inference (Fig 1, S1 and S2 Figs).

We recovered the *Huicundomantis* subgenus as monophyletic with strong support (bootstrap values = 99.5%; posterior probabilities = 1) in both ML and BI analyses similarly to the results of Páez & Ron [23]. The main difference between our phylogram and the one from [23] is in the position of the branch of *P*. *hampatusami* and an undescribed species (UCS3), as well as the position of several unresolved branches such as *P*. *jimenezi*, *P*. *phoxocephalus*, *P*. *totoroi* and *P*. *tinguichaca*, probably due to the different genes used in the analyses (Fig 1). Our tree has a very similar topology with the one presented in [7]. *Pristimantis lojanus* sp. nov. is part of the strongly supported *P*. *phoxocephalus* group (sensu [23]) and its sister species is *P*. *torresi* (Fig 1). Uncorrected *p*-genetic distances for the gene *16S* between *P*. *lojanus* sp. nov. and all its relatives range from 5.0% to 10.9% (S2 Table). The intraspecific genetic distances of the revised specimens did not surpass 0.3%. Based on the large genetic distances to it congeners, advertisement call and morphological differences we describe this new species below.

### Taxonomic treatment

Class Amphibia Linnaeus, 1758

Order Anura Fischer von Waldheim, 1813

Superfamily Brachycephaloidea Günther, 1858

Family Strabomantidae Hedges, Duellman, and Heinicke, 2008

Genus *Pristimantis* Jiménez de la Espada, 1870

### *Pristimantis lojanus* sp. nov. Székely, Székely, Ordóñez-Delgado, Armijos-Ojeda, and Vörös

urn:lsid:zoobank.org:act:39A5A2B9-918E-4DDA-950B-D5F7B51ACE93

(Figs 2–10)

**Fig 2:**
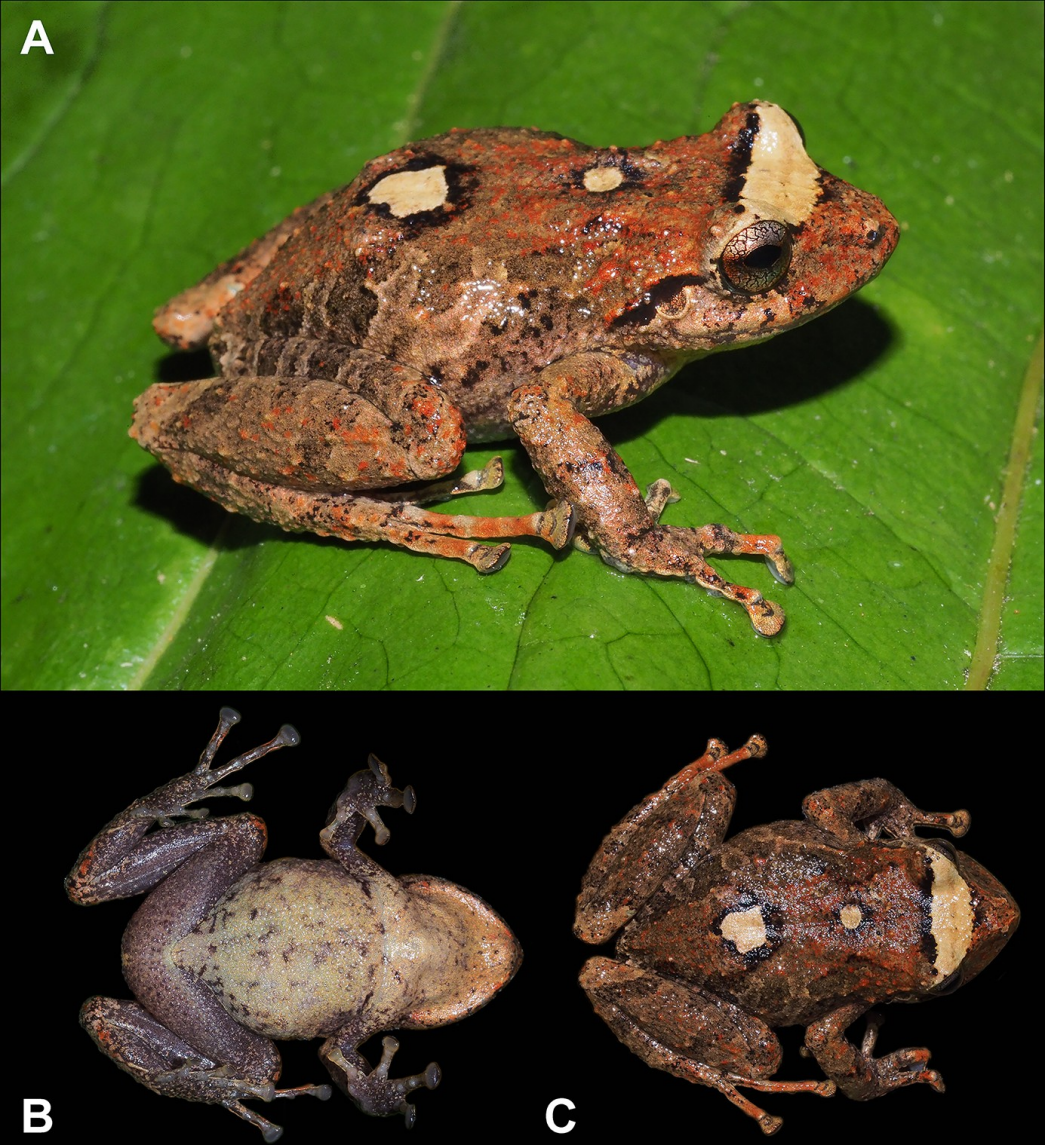
Holotype of *Pristimantis lojanus* sp. nov. (MUTPL 178, adult female), SVL 35.2 mm, in life. A. Dorsolateral view; B. Ventral view; C. Dorsal view.

**Fig 3:**
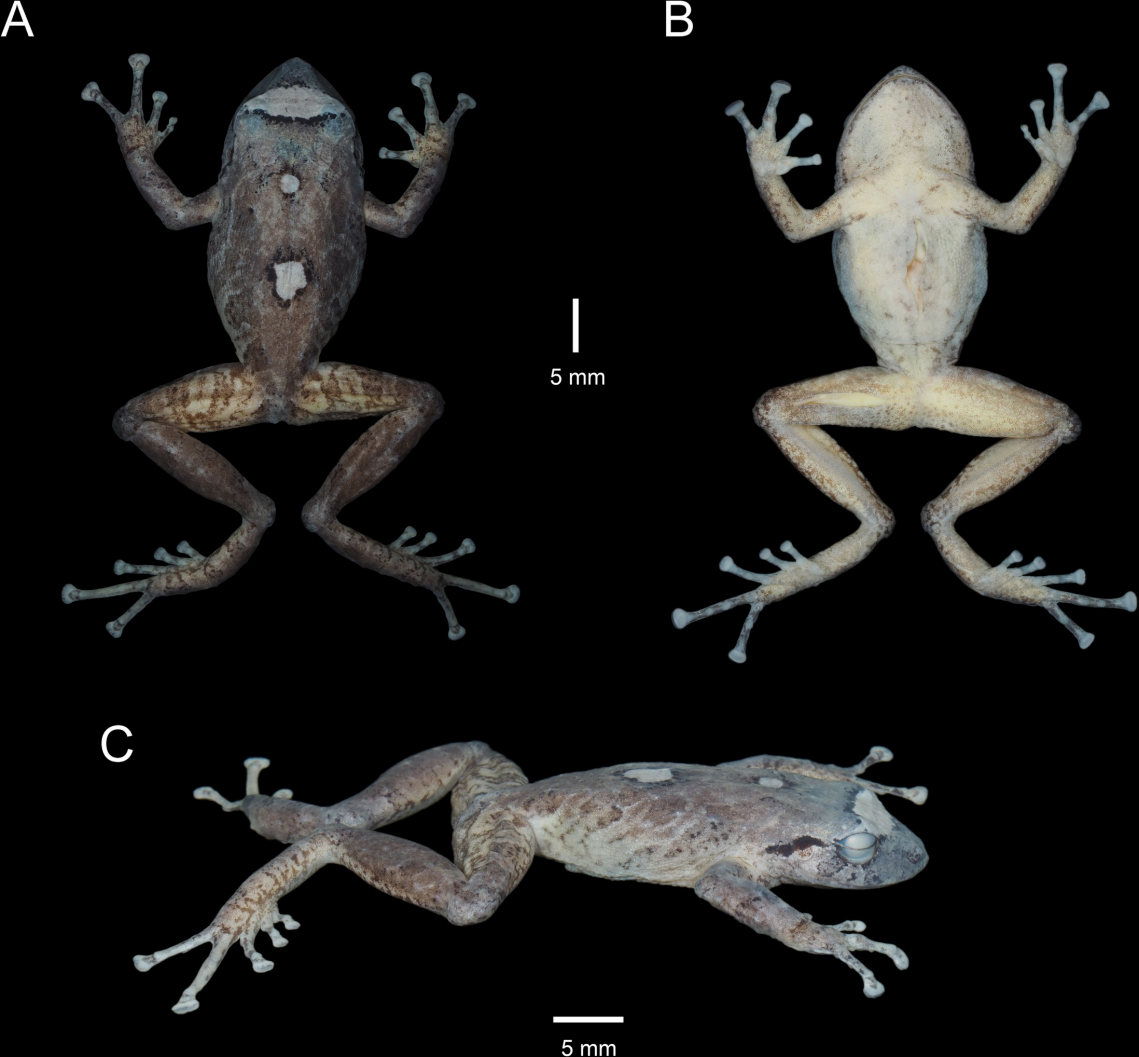
Holotype of *Pristimantis lojanus* sp. nov. (MUTPL 178, adult female) in preservative. A. Dorsal view; B. Ventral view; C. Lateral view.

**Fig 4:**
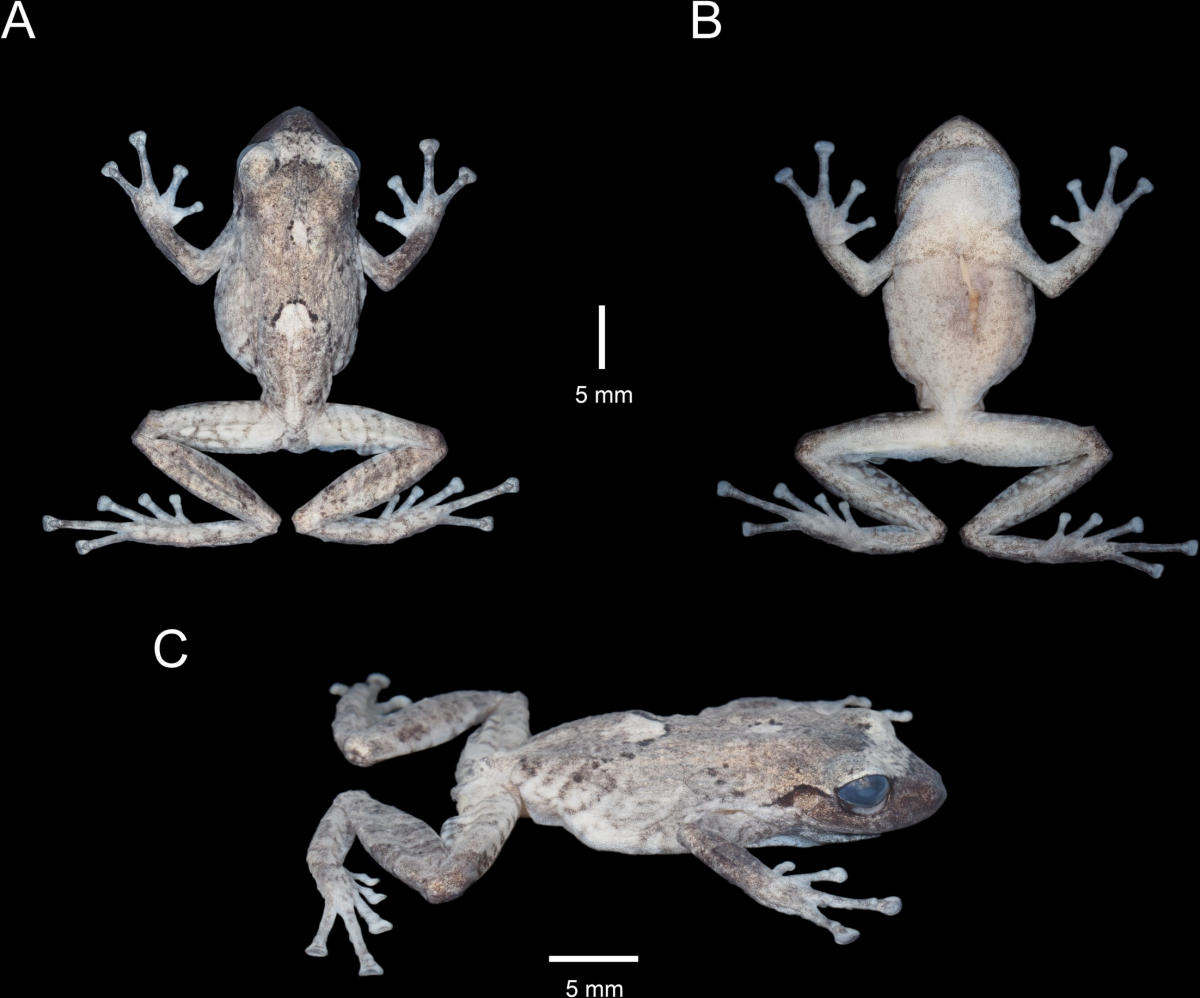
Paratype of *Pristimantis lojanus* sp. nov. (MUTPL 1030, adult male), SVL 28.5 mm, in preservative. A. Dorsal view; B. Ventral view; C. Lateral view.

**Fig 5:**
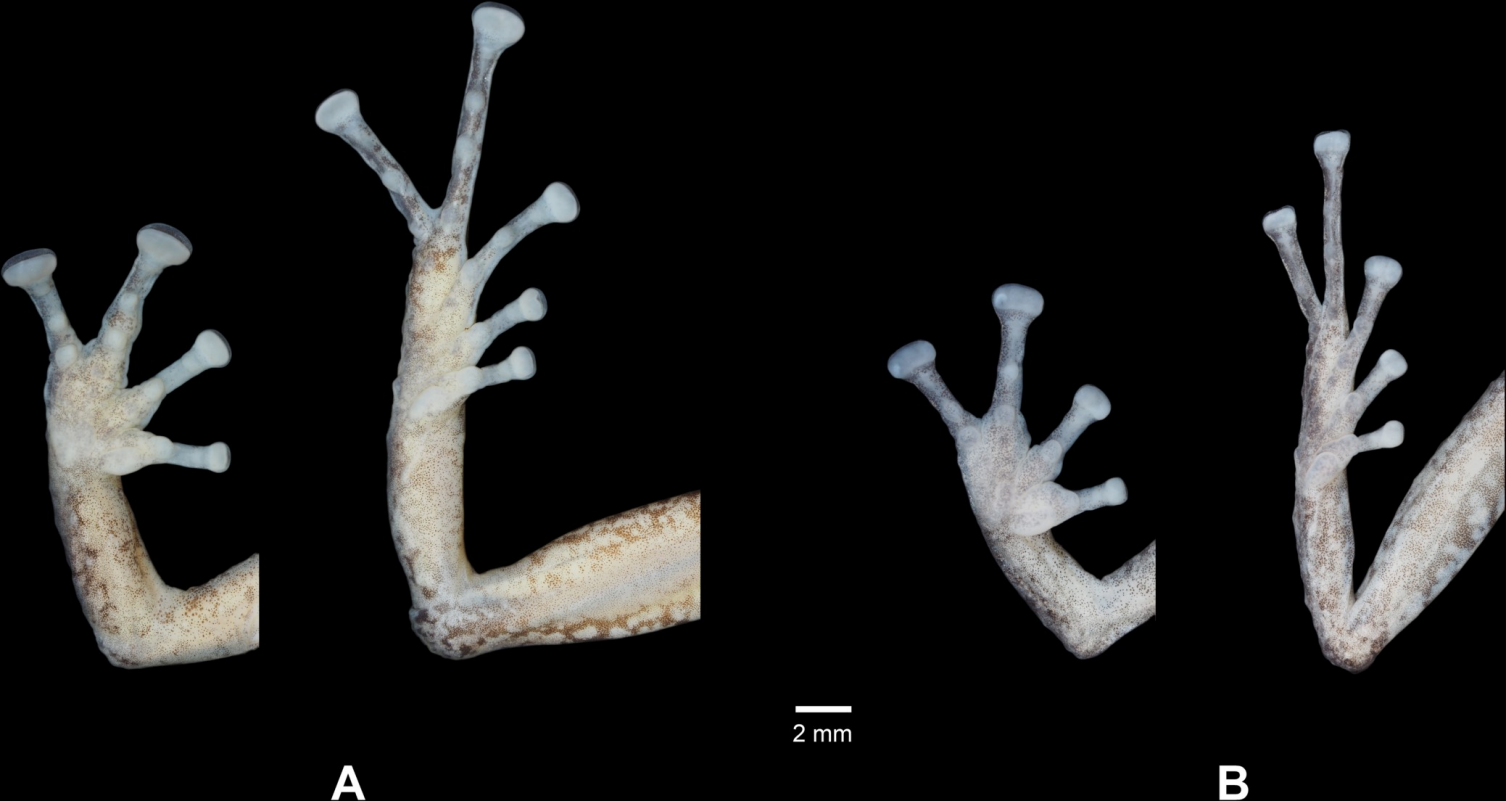
Palmar view of hand and plantar view of foot of (A) holotype of *Pristimantis lojanus* sp. nov. (MUTPL 178, adult female) and (B) paratype (MUTPL 1030, adult male) in preservative.

**Fig 6:**
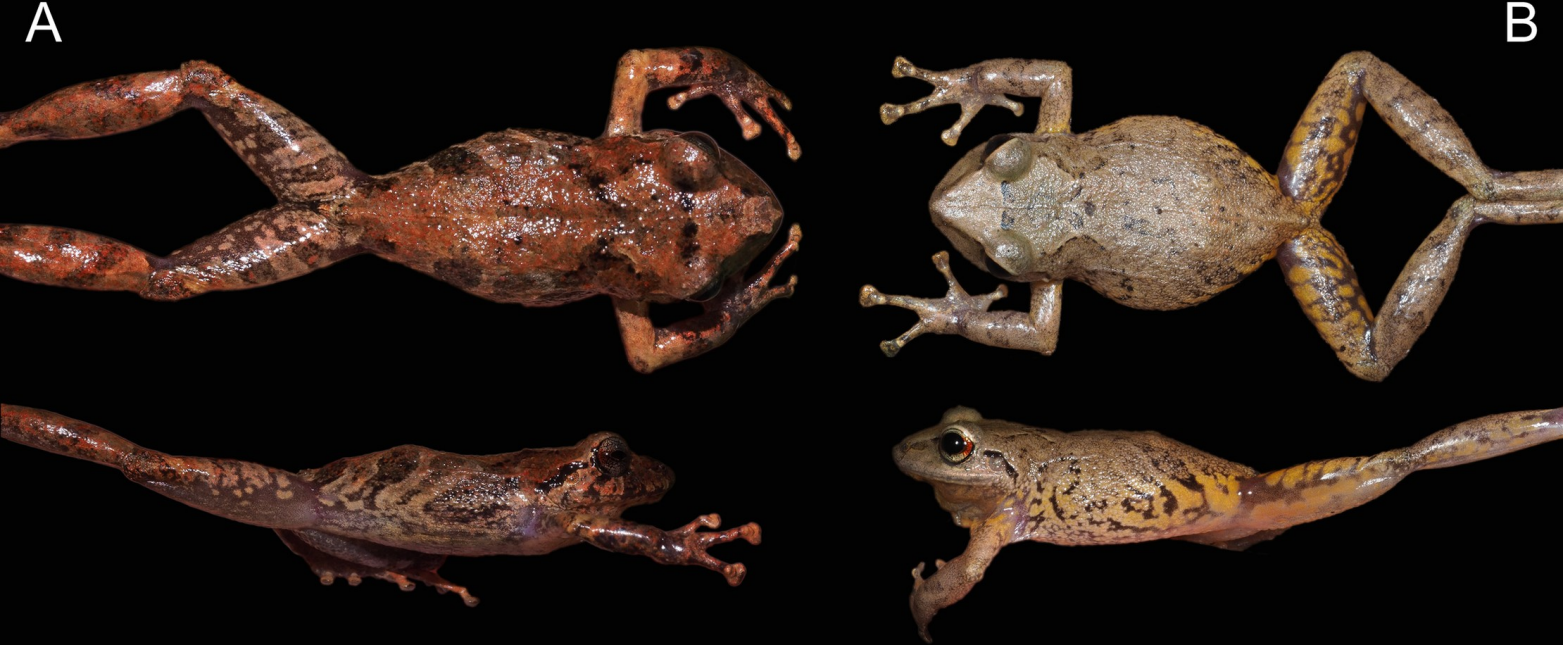
Morphological differences between (A) *Pristimantis lojanus* sp. nov. (male paratype MUTPL 179) and (B) *P*. *torresi* (male, MUTPL 996): no markings in axilla, groin or on concealed limb surfaces vs. yellow markings, and iris bronze vs. iris much lighter, golden. Note: not all *P*. *torresi* individuals have these yellow markings however no *P*. *lojanus* sp. nov. individuals were found with similar markings.

**Fig 7:**
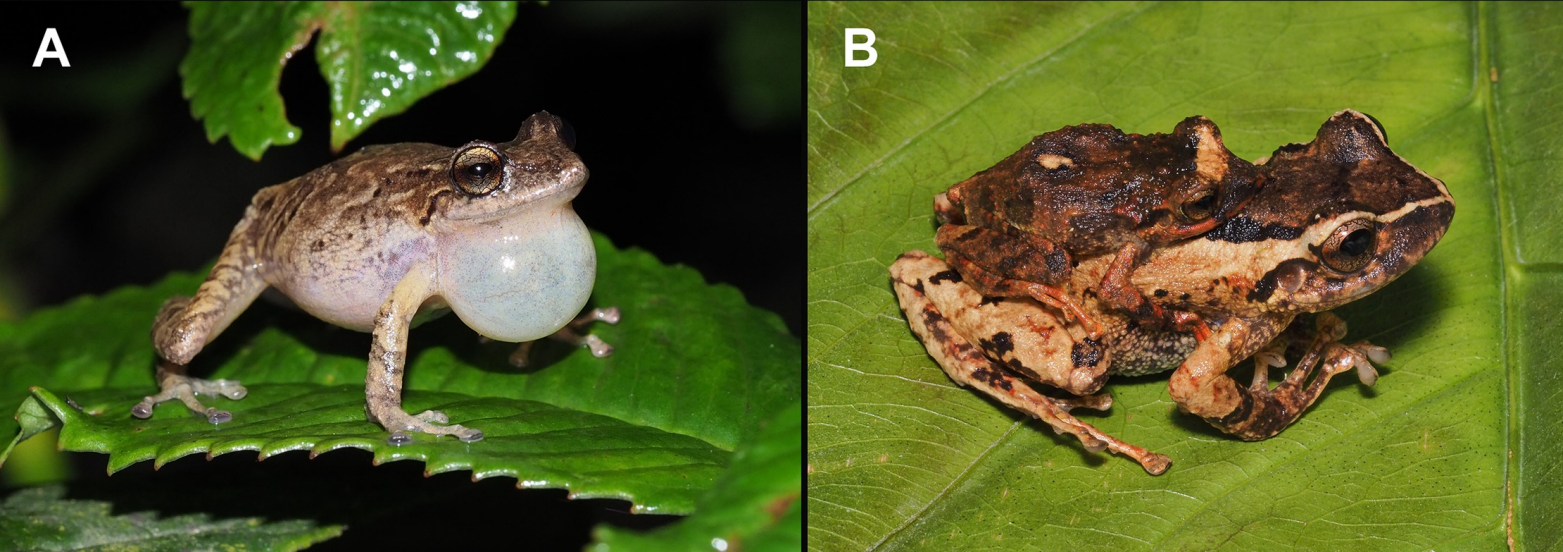
Sexual dimorphism in *Pristimantis lojanus* sp. nov. A. calling male with the inflated subgular vocal sac (not collected, Cerro Sacama) and B. pair in amplexus (paratypes MUTPL 191 and MUTPL 192, Cristal).

**Fig 8:**
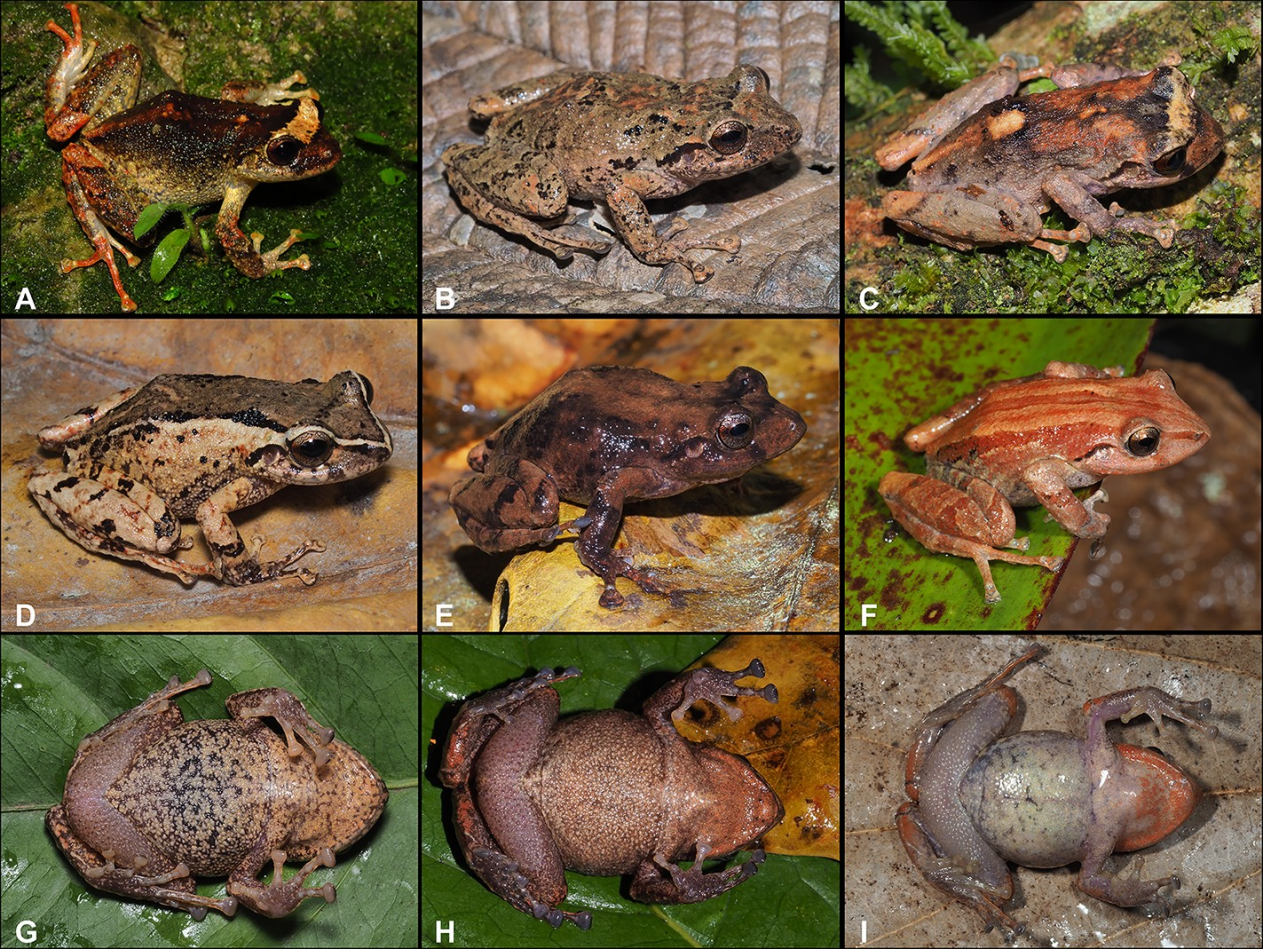
Color variation in females of *Pristimantis lojanus* sp. nov. in life. A. Not collected, Loja city, Parque Universitario de Educación Ambiental y Recreación “Francisco Vivar Castro”; B. Paratype, (MUTPL 176), SVL 37.8 mm, Loja city, Quebrada Volcan; C. Not collected, Cristal; D and G Paratype, (MUTPL 192), SVL 44.3 mm, Cristal; E and H Paratype, (MUTPL 177), SVL 42.1 mm, Loja city, Quebrada San Simon; F and I Paratype, (MUTPL 935), SVL 34.9 mm, Cerro Sacama; A–F Dorsolateral views; G–I Ventral views.

**Fig 9:**
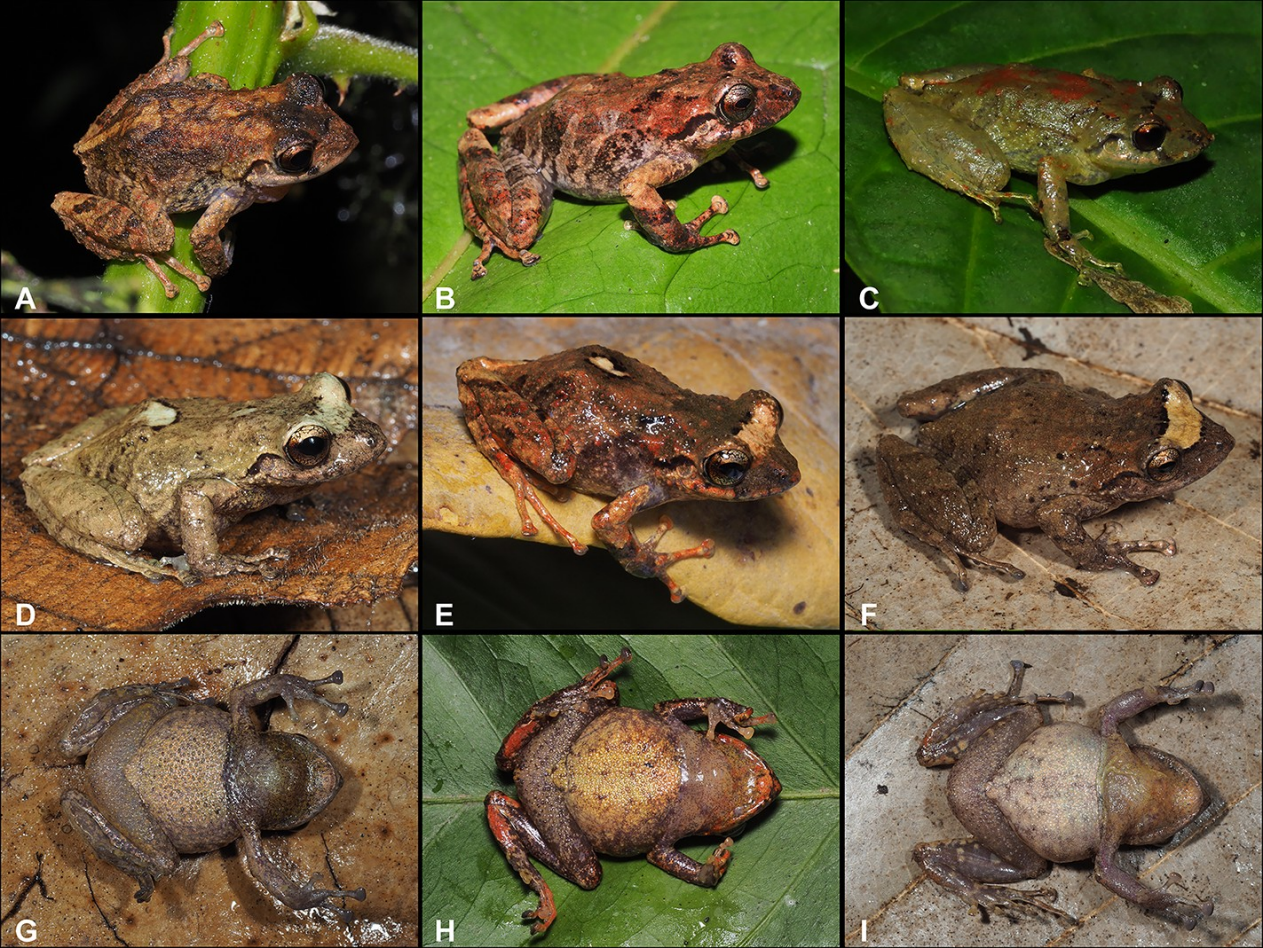
Color variation in males of *Pristimantis lojanus* sp. nov. in life. A. Not collected, Loja city, Parque Universitario de Educación Ambiental y Recreación “Francisco Vivar Castro” (PUEAR); B. Paratype, (MUTPL 179), SVL 27.1 mm, Loja city, Quebrada El Carmen; C. Not collected, PUEAR; D and G Paratype, (MUTPL 1030), SVL 28.5 mm, Loja city, Cerro Chiriaco; E and H Paratype, (MUTPL 191), SVL 26.2 mm, Cristal; F and I Paratype, (MUTPL 936), SVL 30.4 mm, Cerro Sacama; A–F Dorsolateral views; G–I Ventral views.

**Fig 10:**
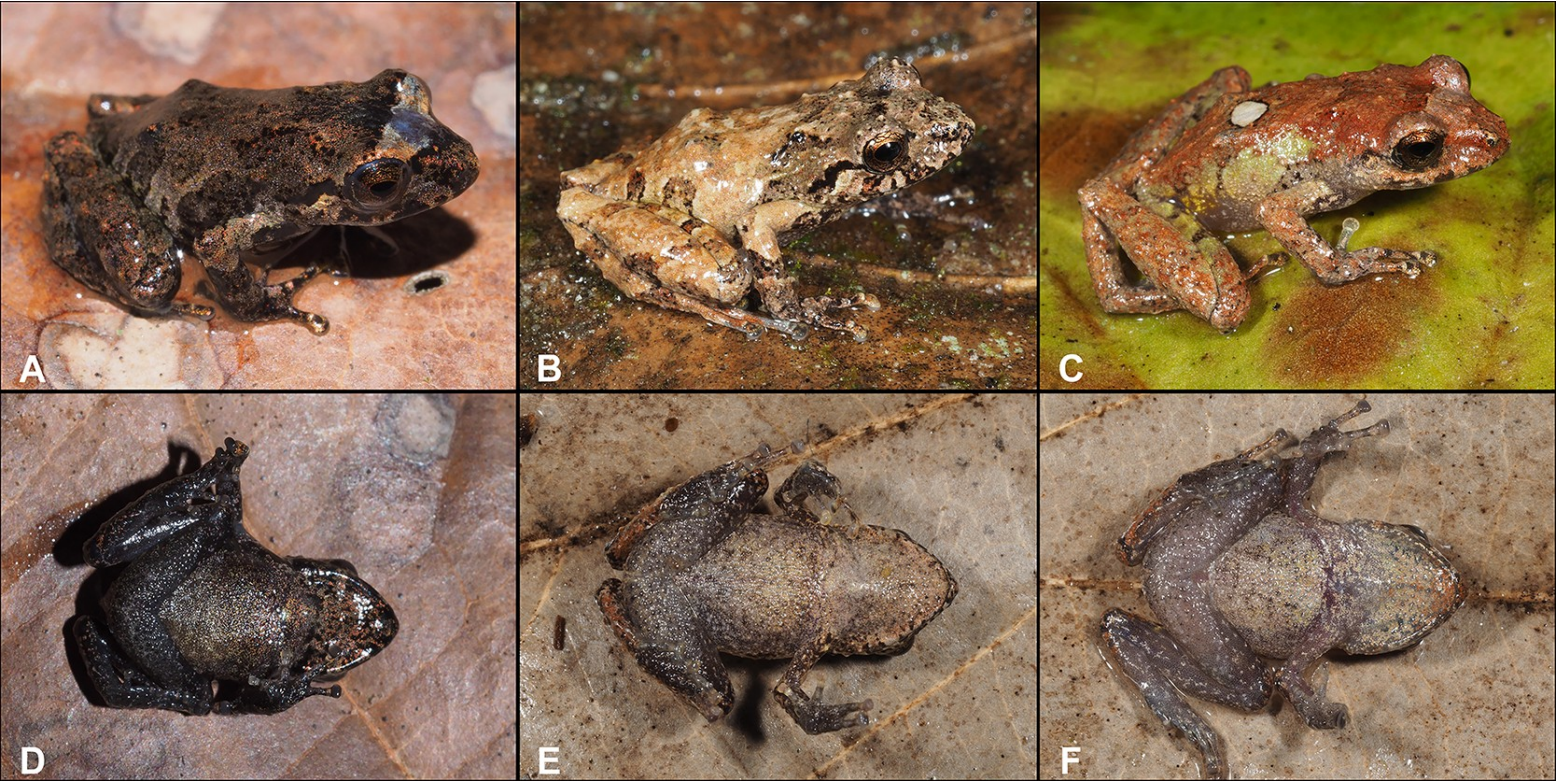
Color variation in juveniles of *Pristimantis lojanus* sp. nov. in life. A and D Paratype, (MUTPL 108), SVL 13.6 mm, Loja city, Quebrada Volcan; B and E Paratype, (MUTPL 628), SVL 18.1 mm, Loja city, Quebrada Quilloyacu; C and F Paratype, (MUTPL 704), SVL 17.8 mm, San Lucas, Acacana. A–C Dorsolateral views; D–F Ventral views.

#### Common English name

Loja Rain Frog

#### Common Spanish name

Cutín de Loja

#### Etymology

The species epithet refers to the type locality, the city of Loja, with the Latin suffix "-*anus*" meaning "belonging to".

#### Holotype

MUTPL 178 (Figs 2, 3 and 5A), an adult female from Ecuador, Loja Province, Loja city, Quebrada El Carmen (4.0432° S, 79.1728° W; datum WGS84), 2268 m above sea level, collected by Paul Székely on 14 July 2016.

#### Paratypes (19: 9 females, 6 males and 4 juveniles)

Ecuador, Loja Province: MUTPL 179, adult male (Fig 9B), collected with the holotype; MUTPL 9, MUTPL 11, MUTPL 12, MUTPL 14, adult females, MUTPL 15, MUTPL 16, adult males, from Loja city, Parque Universitario de Educación Ambiental y Recreación “Francisco Vivar Castro” (3.9836° S, 79.1307° W; 2204 m), collected by Diego Armijos on 25 October 2012; MUTPL 108, juvenile (Fig 10A and 10D), from Loja city, Quebrada Volcan (3.9512° S, 79.1647° W; 2266 m), collected by Diego Armijos on 23 May 2016; MUTPL 176, adult female (Fig 8B), from Loja city, Quebrada Volcan (3.9512° S, 79.1647° W; 2266 m), collected by Paul Székely, Diana Székely and Diego Armijos on 07 July 2016; MUTPL 177, adult female (Fig 8E and 8H), from Loja city, Quebrada San Simon (4.0427° S, 79.1736° W; 2271 m), collected by Diego Armijos and Daniela Sánchez on 14 July 2016; MUTPL 191, adult male (Fig 9E and 9H), MUTPL 192, adult female (Fig 8D and 8G), from Cristal (4.1248° S, 79.1928° W; 2016 m), collected by Paul Székely, Diana Székely and Diego Armijos on 28 January 2017; MUTPL 280, juvenile, from Cristal (4.1227° S, 79.1994° W; 1941 m), collected by Paul Székely, Diana Székely and Santiago Hualpa on 13 June 2017; MUTPL 628 (SC 759), juvenile (Fig 10B and 10E), from Loja city, Quebrada Quilloyacu (4.0710° S, 79.2029° W; 2278 m), collected by Diego Armijos on 11 September 2019; MUTPL 704 (SC 760), juvenile (Fig 10C and 10F), from San Lucas, Acacana (3.7101° S, 79.2545° W; 2736 m), collected by Diego Armijos on 25 September 2019; MUTPL 923 (SC 1134), adult female, from the road to the Cajanuma entrance of the Podocarpus National Park (4.1123° S, 79.1820° W; 2660 m), collected by Paul Székely and Diana Székely on 01 July 2020; MUTPL 935 (SC 1138), adult female (Fig 8F and 8I), MUTPL 936 (SC 1139), adult male (Fig 9F and 9I), from Cerro Sacama (3.8992° S, 79.2577° W; 2572 m), collected by Paul Székely, Diana Székely and Diego Armijos on 16 October 2020; MUTPL 1030 (SC 1179), adult male (Figs 4, 5B, 9D and 9G), from Loja city, Cerro Chiriaco (4.0311° S, 79.2402° W; 2731 m), collected by Diego Armijos, Paul Székely and Diana Székely on 26 February 2021.

#### Diagnosis

We assign this species to *Pristimantis* based on phylogenetic evidence (Fig 1) and on the general morphological similarity to other members of the genus. *Pristimantis lojanus* is a medium sized species, distinguished by the following combination of traits: (1) skin on dorsum finely tuberculated with scattered larger tubercles (in life the skin tuberculated texture is more evident); skin on venter coarsely areolate to areolate; discoidal fold weak; dorsolateral folds absent; low middorsal fold present; (2) tympanic annulus prominent and tympanic membrane differentiated, its length about 45% of the length of eye; supratympanic fold present, concealing the upper and posterior margin of the tympanum; (3) snout acuminate with a vertical keel in dorsal view, rounded or subacuminate and inclined posteroventrally in profile; canthus rostralis weakly concave in dorsal view, rounded in profile; (4) upper eyelid bearing several small tubercles, similar in size and shape with the ones from the dorsum, about 80% IOD in females and 90% IOD in males; cranial crests absent; (5) dentigerous processes of vomers prominent, oblique, ovoid or triangular, separated medially by distance lower than the width of processes; each processes bearing 4 to 7 teeth; (6) males with a large subgular vocal sac and round vocal slits; nuptial pads present; (7) Finger I shorter than Finger II; discs on fingers broadly expanded, truncate; circumferential grooves present; (8) fingers bearing lateral fringes (trait more evident in life); subarticular tubercles prominent; supernumerary palmar tubercles present; palmar tubercle usually partially divided into a larger (inner) and a smaller (outer) tubercles; thenar tubercle elliptical, larger than the inner palmar tubercle; (9) ulnar tubercles present; (10) heel with several small, rounded tubercles; outer edge of tarsus with a row of small tubercles; inner tarsal fold present; (11) inner metatarsal tubercle broadly ovoid, about 4x or 5x the size of subconical (in profile) outer metatarsal tubercle; supernumerary plantar tubercles present; (12) toes bearing broad lateral fringes (trait more visible in life); webbing basal; Toe V much longer than Toe III; discs on toes broadly expanded, truncate, about same size as those on fingers; circumferential grooves present; (13) in life, dorsum and flanks of various shades of brown or reddish brown, with or without whitish spots or blotches, with or without whitish interorbital bars; venter cream, whitish or yellowish, with or without dark flecks and blotches; no markings in axilla, groin or on concealed limb surfaces; iris bronze with fine black reticulations and a median, horizontal read streak; (14) SVL 28.5–44.3 mm in adult females (34.6 ± 5.39 SD, *n* = 10) and 26.2–30.4 mm in adult males (28.1 ± 1.43 SD, *n* = 6).

#### Comparison with similar species

*Pristimantis lojanus* is morphologically similar to the species from the *P*. *phoxocephalus* group (sensu [23]), but it can be distinguished from all the resembling species. *Pristimantis lojanus* is most similar to its closest relative *P*. *torresi* (*p*-genetic distance between 5.0% and 8.4%; S2 Table), from which it differs by the following: slightly larger size (females SVL up to 44.3 mm vs. females SVL up to 39.5 mm; males SVL up to 30.4 mm vs. males SVL up to 30.0 mm), dorsum finely tuberculate with scattered larger tubercles (vs. shagreen dorsum in *P*. *torresi*), flanks without longitudinal lateral folds (vs. flanks with longitudinal lateral folds on anterior half), no markings in axilla, groin or on concealed limb surfaces (vs. many individuals with yellow, cream or orangey-yellow markings; Fig 6) and iris bronze (vs. iris much lighter, golden to beige; Figs 6, 8 and 9). However, the most evident difference between the two species is the advertisement call (see call descriptions below).

Somewhat similar are its northern, distant relatives that also have acuminate snout with a fleshy keel: *P*. *atillo*, *P*. *jimenezi*, *P*. *phoxocephalus*, *P*. *teslai*, *P*. *totoroi*, and *P*. *verrucolatus*. From these, *P*. *teslai* resembles most due to its tuberculate dorsum and darker iris, however *P*. *lojanus* is bigger (males SVL up to 30.4 mm vs. males SVL up to 27.3 mm; females are unknown in *P*. *teslai*), has finely tuberculate dorsum with scattered larger tubercles (vs. tuberculate dorsum, with some prominent tubercles) and bronze iris (vs. copper iris). *Pristimantis lojanus* can be distinguished from the others by the following characters (characters of *P*. *lojanus* in parenthesis): *P*. *atillo* has a shagreen dorsum with or without scattered small tubercles (vs. dorsum finely tuberculated with scattered larger tubercles) and usually has orange groins and black dots on the flanks (vs. no markings in axilla, groin or flanks); *P*. *jimenezi* has a shagreen dorsum with or without scattered small tubercles (vs. dorsum finely tuberculated with scattered larger tubercles), groins and posterior surfaces of thighs with small light brown to yellow spots (vs. no markings in axilla, groin or flanks) and iris reddish copper (vs. iris bronze); *P*. *phoxocephalus* has groins and concealed surfaces of thighs yellow with dark brown to black reticulations (vs. no markings in axilla, groin or on concealed limb surfaces); *P*. *totoroi* has a shagreen dorsum with or without scattered small tubercles (vs. dorsum finely tuberculated with scattered larger tubercles) and iris golden (vs. iris bronze); *P*. *verrucolatus* has a shagreen dorsum with or without scattered tubercles (vs. dorsum finely tuberculated with scattered larger tubercles) and with thick lateral folds, warts and larger tubercles on the flanks (vs. dorsolateral folds, warts and larger tubercles on the flanks absent).

#### Description of the holotype

Adult female (MUTPL 178; Figs 2, 3 and 5A), head narrower than body, wider than long, head length 81% of head width, head width 39% of SVL; head length 32% of SVL; snout moderately long (snout to eye distance 17% of SVL), acuminate with a vertical keel in dorsal view and subacuminate and inclined posteroventrally in profile (Figs 2 and 3); canthus rostralis weakly concave in dorsal view, rounded in profile; loreal region slightly convex; eye diameter smaller than eye-nostril distance; nostrils slightly protuberant, oriented posteriorly; lips not flared; cranial crests absent; upper eyelid bearing several small tubercles (three larger than the others), width of upper eyelid 74% of IOD; tympanic annulus prominent and tympanic membrane differentiated; thick supratympanic fold present, concealing the upper and posterior margin of the tympanum (Fig 2A); diameter of tympanum 46% of the length of eye; two larger, rounded postrictal tubercles surrounded by several smaller tubercles; choanae large, round, not concealed by palatal shelf of maxillary arch; dentigerous processes of vomers prominent, slightly larger than the choanae, oblique, situated posterior and median to choanae, triangular in outline, not separated medially, each processes bearing 5 to 6 teeth; tongue longer as wider, slightly notched posteriorly, posterior half not adherent to floor of mouth.

Skin on dorsum finely tuberculated with scattered larger tubercles (in life the skin tuberculated texture was more evident, Figs 2A, 2C and 3A); thin, low middorsal fold starting at tip of snout and ending at cloaca; dorsolateral folds absent; skin on chest, belly, and ventral surfaces of thighs coarsely areolate; thoracic and discoidal folds weak (Figs 2B and 3B); cloacal region bordered ventrally by several small tubercles.

Ulnar tubercles present; outer palmar tubercle prominent, partially divided into a larger (inner) and a smaller (outer) tubercles; thenar tubercle elliptical, larger than the inner palmar tubercle; subarticular tubercles prominent, round and subconical in section; supernumerary palmar tubercles rounded, large, slightly smaller than subarticular tubercles; fingers bearing lateral fringes (trait more evident in life); relative length of fingers I < II < IV < III; discs on fingers broadly expanded, truncate; all fingers bearing pads well defined by circumferential grooves (Fig 5A).

Hindlimbs long, slender; tibia length 51% of SVL; foot length 44% of SVL; heel with several small, rounded tubercles; outer edge of tarsus with a row of small, inconspicuous, tubercles (trait more visible in life); inner edge of tarsus bearing a long fold; inner metatarsal tubercle broadly ovoid, about 4x round and subconical (in profile) outer metatarsal tubercle; subarticular tubercles prominent, round and subconical in section; plantar supernumerary tubercles rounded, smaller than subarticular tubercles; toes bearing broad lateral fringes (trait more visible in life); webbing basal; discs on toes broadly expanded, truncate, about same size as those on fingers; toes with ventral pads well defined by circumferential grooves (Fig 5A); relative length of toes I <II < III < V < IV; Toe V much longer than Toe III (tip of Toe III reaches the middle of the penultimate subarticular tubercle on Toe IV, tip of Toe V extends beyond the distal edge of distal subarticular tubercle on Toe IV).

#### Coloration of holotype

In life (Fig 2): dorsum and snout reddish brown, flanks and dorsal surfaces of femurs light brown with dark brown bars; two black bordered, yellowish white blotches on dorsum (one larger and one smaller) and a wide interorbital bar with the same coloration; black supratympanic bars; dorsal surfaces of hindlimbs, arms, toes and fingers covered with reddish brown irregular markings; venter and throat yellowish white, ventral surfaces of hindlimbs and arms grey; iris bronze with a dark red, median horizontal streak and with black reticulations.

In preservative (Fig 3): dorsum brownish gray, flanks and dorsal surfaces of femurs yellowish white with brownish gray bars; the two black bordered blotches on the back and the wide interorbital bar became white; black supratympanic bars; venter, throat, ventral surfaces of hindlimbs and arms yellowish white.

**Measurements of holotype (in mm):** SVL 35.2; HW 13.7; HL 11.1; IOD 4.2; IND 2.7; EW 3.1; ED 3.3; EN 3.6; snout to eye distance 5.8; FL 15.7; TL 17.9; FoL 15.3; HaL 9.8; Finger I length 4.6.

**Body mass of holotype:** 3.09 g.

#### Variation

Morphometric variation is shown in Table 1. This species displays an evident sexual dimorphism, the females being significantly larger than the males (Fig 7). Both females and males vary greatly in the dorsal coloration, the majority of encountered individuals displaying various shades of brown or reddish brown, with or without whitish spots or blotches, with or without whitish interorbital bars. Female MUTPL 192 (Fig 8D) had a wide, dark brown middorsal band and contrasting yellowish white flanks, female MUTPL 935 (Fig 8F) was almost red (dark reddish brown with lighter stripes), and one male (Fig 9F) was greenish with red spots on the dorsum. Only one individual (MUTPL 760, Fig 10A), from the many animals encountered by us, displayed some yellowish markings in the groin and concealed limb surfaces (but not like the widespread markings usually encountered in *P*. *torresi*). As for the iris, female MUTPL 177 (Fig 8E) and male MUTPL 179 (Fig 9B) had an atypically light, whitish bronze coloration. In all specimens, the supratympanic fold was black.

**Table 1:** Body mass (in grams), measurements (in mm) and morphological proportions (in percentages) of adult females and males of *Pristimantis lojanus* sp. nov.

Character	females (*n* = 10)	males (*n* = 6)
Body mass (BM)	3.77 ± 1.57 (1.76–6.02)*	1.54 ± 0.25 (1.34–1.90)**
Snout-vent length (SVL)	34.6 ± 5.39 (28.5–44.3)	28.1 ± 1.43 (26.2–30.4)
Head width (HW)	12.7 ± 1.79 (10.1–15.6)	10.0 ± 0.65 (9.2–11.0)
Head length (HL)	11.2 ± 1.44 (9.6–13.6)	9.2 ± 0.77 (8.1–10.4)
Interorbital distance (IOD)	3.8 ± 0.59 (3.0–4.5)	2.9 ± 0.38 (2.7–3.7)
Internarial distance (IND)	2.6 ± 0.31 (2.2–3.1)	2.1 ± 0.10 (2.0–2.3)
Upper eyelid width (EW)	3.0 ± 0.61 (2.3–4.3)	2.6 ± 0.20 (2.2–2.7)
Eye diameter (ED)	3.4 ± 0.50 (3.0–4.5)	3.1 ± 0.27 (2.8–3.5)
Eye-nostril distance (EN)	3.7 ± 0.59 (2.8–4.8)	2.9 ± 0.28 (2.5–3.3)
Tympanum diameter (TD)	1.6 ± 0.24 (1.2–2.0)	1.3 ± 0.18 (1.1–1.4)
Femur length (FL)	15.2 ± 1.69 (12.7–18.2)	12.6 ± 0.75 (11.8–13.9)
Tibia length (TL)	17.3 ± 1.80 (14.6–20.5)	14.1 ± 0.48 (13.8–15.0)
Foot length (FoL)	15.0 ± 1.93 (12.1–18.0)	12.5 ± 1.18 (11.0–14.0)
Hand length (HaL)	9.4 ± 1.22 (7.7–11.3)	7.6 ± 0.52 (6.8–8.3)
HW/SVL	34.9–38.9	34.4–37.0
HL/SVL	30.7–35.5	30.9–34.2
HL/HW	81.0–95.0	88.0–95.9
EN/HL	29.2–35.3	29.7–32.2
ED/HL	26.5–35.1	28.8–37.2
EW/IOD	62.8–95.6	73.0–96.4
EN/ED	90.3–123.3	85.7–110.0
TD/ED	40.0–63.3	37.1–46.7
FL/SVL	40.6–46.8	43.1–45.7
TL/SVL	45.1–55.6	48.8–53.1
FoL/SVL	40.6–45.1	42.0–48.2
HaL/SVL	25.4–29.8	26.0–27.5

Values are given as mean ± SD (range). Female body mass includes eggs.

* *n* = 6

** *n* = 4.

#### Advertisement cal

**Fig 11:**
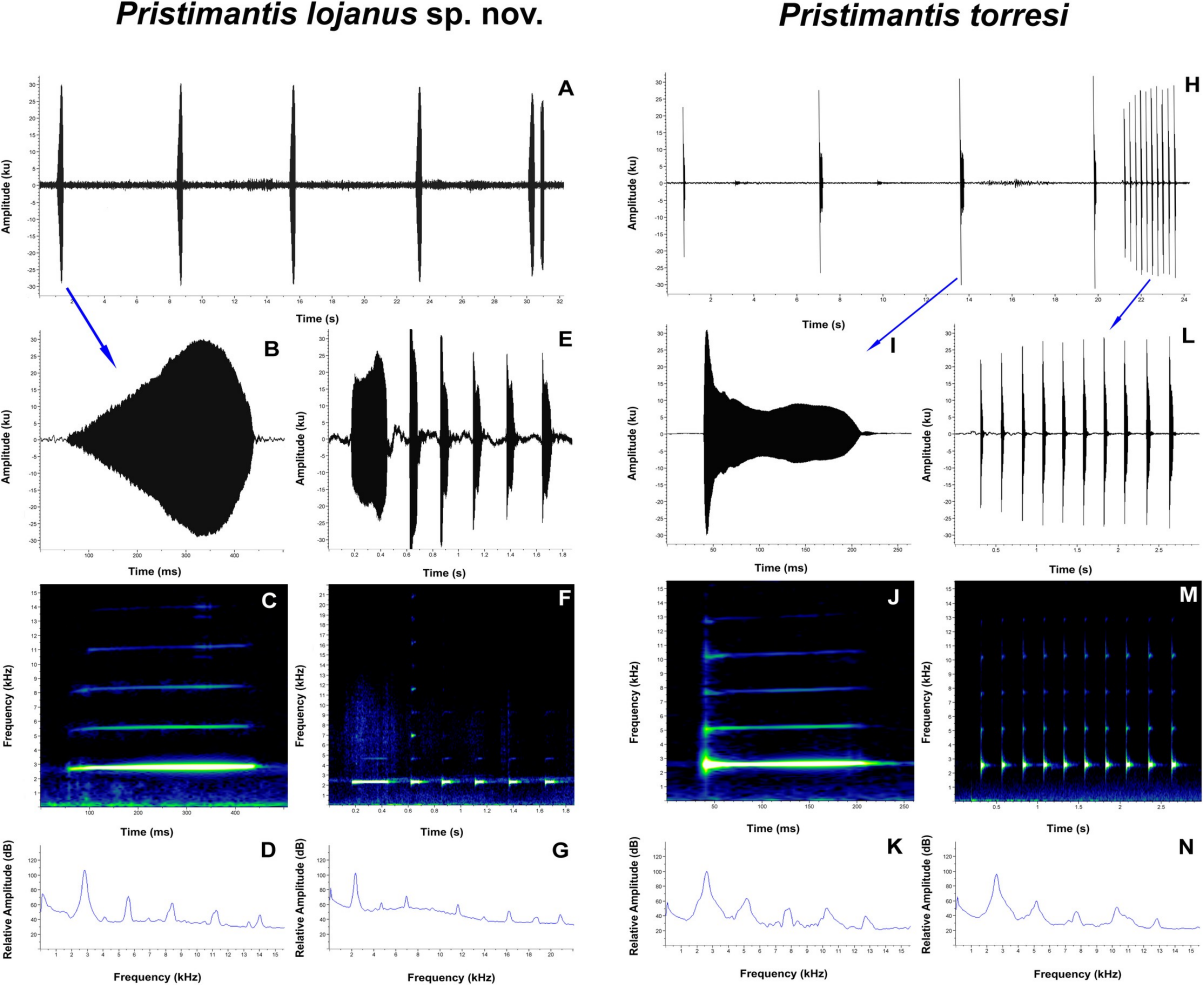
Advertisement calls of *Pristimantis lojanus* sp. nov. (A–G) and *P*. *torresi* (H–N). A. Oscillogram of a 5 calls section of the call series, with single and double-noted (last one) calls; B and I Oscillograms of single-noted calls; C and J Spectrograms of single-noted calls; D and K Power spectrums of single-noted calls; E and L Oscillograms of multi-noted calls; F and M Spectrograms of multi-noted calls; G and N Power spectrums of multi-noted calls; H. Oscillogram of a 5 calls section of the call series, with single and multi-noted (last one) calls; A–D FUTPL-A 252; E–G FUTPL-A 249; H–N MUTPL 996, FUTPL-A 256. All spectrograms at Hanning window function, 512 bands resolution. See text for details.

For the description of the *P*. *lojanus* call, we analyzed 7 recordings, two from Loja city, Parque Universitario de Educación Ambiental y Recreación “Francisco Vivar Castro” (from 2015 and 2016), one from Abra de Zamora (2018), one from Cerro Sacama (2020), and three from Loja city, Cerro Chiriaco (2021). Descriptive statistics of the acoustic variables are provided in Table 2 (the detailed information of each of the separate recordings is presented in the S3 Table). *Pristimantis lojanus* has an advertisement call characterized by a call series composed by whistles repeated over a period of time, somewhat similar to the calls of *P*. *balionotus*, *P*. *jimenezi*, or *P*. *verrucolatus* (Fig 11A–11G). The calls are composed usually by one, but sometimes two notes (frequency modulated tonal sounds). The calls are characterized by a mean duration of 0.367 s, a mean inter-call interval of 7.007 s and a mean call rate of 7.80 calls/min (Table 2). In the case of the double-noted calls (Fig 11A), the call duration was 0.797–1.460 s (0.937 ± 0.192, *n* = 10) (S3 Table), the first note being longer than the second: 0.347–0.417 s (0.370 ± 0.021, *n* = 10) vs. 0.136–0.225 s (0.176 ± 0.028, *n* = 10). The mean dominant frequency of the call was 2598.0 Hz, with a mean 90% bandwidth of 2506.3–2674.3 Hz (Table 2). The fundamental frequency is not recognizable, but usually 3 to 8 harmonics are visible.

**Table 2:** Quantitative description of the advertisement calls (mean ± SD, range and *n*) of *Pristimantis lojanus* sp. nov. and *P*. *torresi*.

	*Pristimantis lojanus* sp. nov.	*Pristimantis torresi*
	(7)	(4)
Notes per call	1–6	1–10
	(usually 1 or 2)	(usually 1)
Call duration (s)	0.367 ± 0.023 (0.314–0.443)	0.229 ± 0.040 (0.103–0.364)
	*n* = 107	*n* = 183
Multi-noted call duration (s)	1.596	0.189 ± 0.026 (0.153–0.237)
		*n* = 14
Inter-call interval (s)	7.007 ± 1.182 (4.131–9.851)	5.370 ± 0.992 (3.318–8.165)
	*n* = 98	*n* = 119
Call rate (calls/min)	7.80 ± 0.476 (7.43–8.76)	10.94 ± 2.033 (8.51–13.09)
	*n* = 7	*n* = 4
Short note duration for multi-noted calls (s)	0.080 ± 0.012 (0.066–0.096)	0.046 ± 0.009 (0.021–0.060)
	*n* = 5	*n* = 97
Inter-note interval for multi- noted calls (s)	0.174 ± 0.014 (0.153–0.185)	0.217 ± 0.018 (0.194–0.288)
	*n* = 5	*n* = 97
Short note (for multi-noted calls) rate (notes/s)	3.94	3.78 ± 0.124 (3.60–4.04)
		*n* = 16
Dominant frequency (Hz)	2598.0 ± 201.660 (2325.6–2842.4)	2446.0 ± 88.252 (2153.3–2584.0)
	*n* = 123	*n* = 221
Frequency 5% (Hz)	2506.3 ± 200.159 (2239.5–2756.3)	2362.1 ± 90.911 (2067.2–2497.9)
	*n* = 123	*n* = 222
Frequency 95% (Hz)	2674.3 ± 193.183 (2411.7–2928.5)	2576.5 ± 90.080 (2325.9–2756.3)
	*n* = 123	*n* = 218

The number of samples (calls of specimens) is given in brackets under the species name.

Similarly to some of the other species of the *P*. *phoxocephalus* group [7], the males can sometimes emit special, multi-noted calls, with up to 6 notes (Fig 11E–11G). These calls probably have some different function compared to the typical advertisement calls, and might be used in the case of social interactions, triggered by the presence of nearby females or competitive males.

In December 2020 we recorded four males of *P*. *torresi*, the sister species of *P*. *lojanus*, in its type locality, Guachanamá, El Apretadero, Southern Ecuador (S3 Table). The advertisement calls are somewhat similar to the one of *P*. *lojanus* and are composed by one-noted calls characterized by a mean duration of 0.229 s, a mean inter-call interval of 5.370 s and a mean call rate of 10.94 calls/min (Fig 11H–11N, Table 2). The mean dominant frequency of the call was 2446.0 Hz, with a mean 90% bandwidth of 2362.1–2576.5 Hz (Table 2). The fundamental frequency is not recognizable, but usually 3 to 6 harmonics are visible.

The calls of these two species can be easily distinguished (even by ear) as they differ significantly in call duration, inter-call interval, call rate, and frequencies (Table 2, Fig 11). Additionally, the multi-note calls emitted by these species are also different. In the case of *P*. *lojanus*, the short notes are produced immediately after one long note (Fig 11E) rather than a separate call from the call series like in the case of *P*. *torresi* (Fig 11H). They differ also in the note duration and inter-note interval (Table 2).

The *P*. *torresi* male MUTPL 996 (FUTPL-A 256, 257) emitted in about 10 minutes 14 multi-noted calls, probably triggered by the presence of two competitive males on the nearby branches. One of these males also responded by producing similar multi-noted calls.

#### Distribution

**Fig 12:**
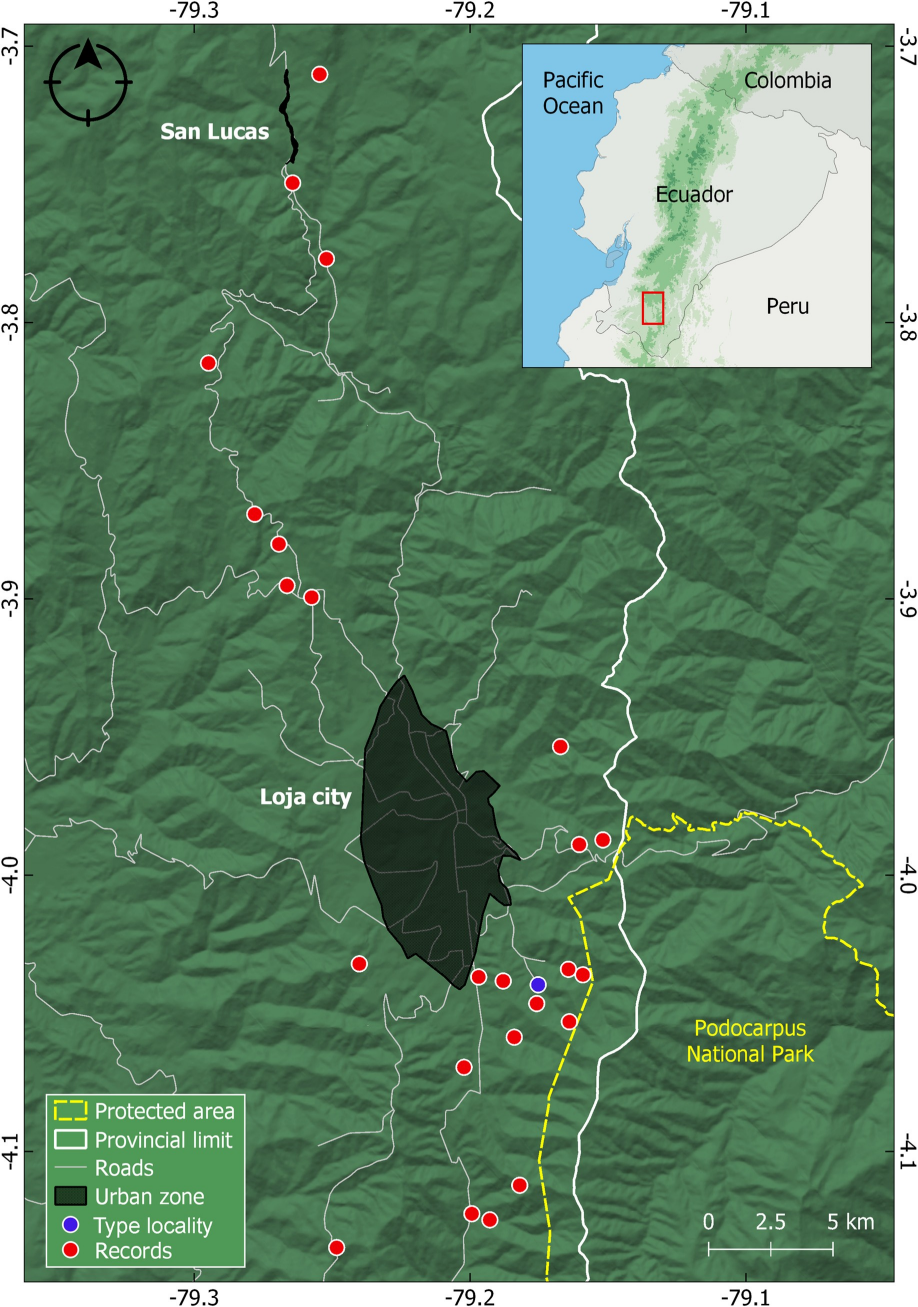
Distribution of *Pristimantis lojanus* sp. nov. in Ecuador. Records are based on specimens deposited at the Museo de Zoología, Universidad Técnica Particular de Loja, Loja, Ecuador (MUTPL) or call recordings.

*Pristimantis lojanus* is known from the city of Loja and its close vicinity (all the streams that flow into the city, but also parks like Parque Universitario de Educación Ambiental y Recreación “Francisco Vivar Castro” and private protected areas like Reserva Madrigal del Podocarpus), as well as from several other localities (Fig 12). We also have confirmed records (with DNA samples or call recordings) from San Lucas and its vicinities, Bosque Servio Aguirre Villamagua, Cerro Sacama, Abra de Zamora, Cajanuma, Cristal, and San Antonio de Paycapamba. The species was encountered at an altitudinal range between 1937 m (Cristal) and 2782 m (Bosque Servio Aguirre Villamagua) a.s.l., in evergreen lower montane forest and evergreen upper montane forest ecosystems (sensu [33]).

#### Natural history

This is a common species sensu [7] (individuals were detected—seen or heard—in the adequate habitat, in large or moderate numbers, on 50–100% of the sampling days/nights). Most specimens were encountered during the night, on the vegetation (shrubs, branches of trees or grassy vegetation) from 10 cm above the ground up to 2–3 m. The majority of the individuals were encountered in habitats close to small streams. Calling males were encountered year-round, but more frequently on rainy nights. All the individuals were encountered in forested areas with some degree of human intervention (secondary forests). The species needs forested areas near the streams, as we could not detect the presence of *P*. *lojanus* on the stream segments from the city where the native vegetation was destroyed.

#### Conservation status

*Pristimantis lojanus* is known from about 25 localities, from an estimated area of about 400 km^2^. Even if this species is common (locally abundant) in the proper habitats, we recommend this species to be categorized as Endangered following the B1ab(iii,iv) + 2ab(iii,iv) IUCN criteria [34] because: (1) its Extent of occurrence (EOO) and Area of occupancy (AOO) are estimated to be less than 400 km^2^; (2) the species distribution is severely fragmented; (3) none of its populations can be found in national protected areas; and (4) its habitats could be severely affected in the near future, as they are situated in the vicinity of densely populated areas.

## Discussion

The distribution of *Pristimantis lojanus* is limited to a relatively small area, mostly included in the Upper Zamora River Basin, an area of approx. 620 km^2^ delimited by the Guagrahuma-Acacana and Cajanuma orographic knots [35, 36]. This region was a tertiary lake basin which opened around Salapa giving birth to the Zamora River [36, 37]. The northernmost records of *P*. *lojanus* are from near San Lucas (Acacana), fieldwork carried out outside this area since 2016 failing to encounter it. In the south, the species reaches Cristal and San Antonio de Paycapamba; further south, around Vilcabamba, *P*. *lojanus* is replaced by a very similar, currently undescribed, species (pers. obs.). Since 2016, all sightings and collected specimens of the *P*. *phoxocephalus* group from the Loja basin can be assigned to *P*. *lojanus*. Due to the similar morphology and based on the available data, we consider that the specimens collected from Loja by Lynch in 1979 [20] and considered to be *P*. *phoxocephalus* are in fact *P*. *lojanus*.

Unfortunately, as the population of the city of Loja increased over the years, the situation of the amphibians from the city and neighboring areas deteriorated. Currently, there are 12 species of amphibians (including *P*. *lojanus*) reported from the city and its surroundings (upper montane forest ecosystems around the city, up to 2700 m, up to the mountain crests and subpáramo habitats). The last record of *Rhinella amabilis* from Loja was in 1971 (the Kansas University Herpetology Collections database, https://biodiversity.ku.edu/herpetology/collections), and we suspect it to be extinct (currently this species is listed as Critically Endangered in the IUCN Red List). Recently, we encountered a toad population inside the city, which turned out to be *R*. *poeppigii* (confirmed by DNA sampling); this is probably a recently introduced population, since this species has a lower altitudinal range (800–1670 m; [38]). Also, there is a large distribution gap between known records in the east, with the species not being detected even in well surveyed areas such as Abra de Zamora and San Francisco Scientific Research Station [7], between 1800–3000 m. The Loja Rocket Frog (*Hyloxalus elachyhistus*) was heard calling in 2019 and 2020 on the heavily transformed banks of Río Zamora, in a couple of locations in the center of the city. However, the population is facing extinction due to contamination and habitat change. For now, *Gastrotheca elicioi* is still common in Loja, but its survival depends on habitat availability, which is threatened by city expansion. In the case of *G*. *lojana*, no recent confirmation (using either molecular or call samples) of the species presence from Loja and its surroundings are available. On the other hand, we encountered *G*. *pseustes* in the northern outskirts of Loja (the species type locality is further north, close to San Lucas), living syntopically with *G*. *elicioi*. *Gastrotheca pseustes* is replaced by *G*. *elicioi* in Loja and further south.

As for the members of Strabomantidae (terrestrial-breeding frogs), there are seven species recorded for the area, three of which having an unclear taxonomic status: *Noblella* aff. *heyeri*, *Pristimantis* aff. *andinognomus*, *P*. *atratus* [20], *P*. aff. *cajamarcensis*, *P*. *cryptomelas* [20], *P*. *lymani* and *P*. *lojanus*. The most common species are *P*. *lymani* (which is the most tolerant to anthropic impact, being able to survive even in the green areas between the buildings), *P*. aff. *cajamarcensis* and *P*. *lojanus*. *Pristimantis* aff. *andinognomus*, *P*. *atratus*, and *P*. *cryptomelas* can be found only at higher elevations, over 2400 m, of the forested areas in the eastern flank of the city.

An unfortunate addition to Loja’s fauna [39] is the Bullfrog, *Lithobates catesbeianus* [40], which inhabits several of the city ponds and can have a negative effect on the survival of the native species, especially of the pond-breeding *G*. *elicioi*. Since 2015 when Cobos et. al. [39] reported the presence of this species from Parque Pucará (4.0127° S, 79.1951° W, 2197 m), we encountered several other ponds in the city with Bullfrog populations: Laguna de la Cruz (4.0540° S, 79.2092° W, 2335 m), Laguna de los Maestros (4.0422° S, 79.2037° W, 2213 m), Laguna del Zoológico (3.9575° S, 79.2173° W, 2014 m), Laguna Santa Bárbara (3.9634° S, 79.2443° W, 2259 m), and Laguna Valle Hermoso (3.9530° S, 79.2438° W, 2221 m). Additionally, a Bullfrog population was detected in Laguna Patonadana (3.7049° S, 79.2469° W, 2839 m), about 3 km north east of San Lucas, were it could jeopardize the survival of the local San Lucas Marsupial Frog (*G*. *pseustes*) population. For this problem, a campaign was initiated to remove this invasive species through the collaboration of Universidad Técnica Particular de Loja, the local government and several local Eco clubs; however, the number of individuals found (e.g. >100/ hour in Parque Pucará) suggests that their control is increasingly difficult. Bullfrog eradication campaigns based on complete removal of individuals using manual methods (hand trapping, netting, electro-shocker, egg-mass removal) are time consuming, expensive and has had limited success in other habitats (e.g., [41–43]), although it can have an important impact on final bullfrog population size. Other methods, such as the use of native predatory fish in artificial ponds might be more effective [44], but there are no studies regarding their feasibility in the Tropical Andes. On the other hand, there is an urgent need for public awareness campaigns aimed at preventing further intentional Bullfrog introductions, as well as continued documentation of its dispersal.

## Supporting information

S1 Fig Maximum likelihood phylogram including outgroup, based on 2339 base pairs of concatenated DNA from *12S*, *16S*, and *RAG-1* gene fragments. (PDF)

S2 Fig Bayesian phylogram including outgroup, based on 2339 base pairs of concatenated DNA from *12S*, *16S*, and *RAG-1* gene fragments. (PDF)

S1 Table Voucher, GenBank accession numbers and locality for the specimens used in the phylogenetic analysis. (DOCX)

S2 Table Uncorrected pairwise distances (%), for the mitochondrial gene *16S* fragment, for the *Huicundomantis* subgenus of *Pristimantis*. (XLSX)

S3 Table Information regarding the call recordings and the bioacoustic measurements for each of the recorded males. Values are given as average ± SD (range) and *n* measured parameter.
(XLSX)

S1 Appendix Additional specimens examined. (DOCX)
