# Supplementary material for: Our unknown neighbor: A new species of rain frog of the genus Pristimantis (Amphibia: Anura: Strabomantidae) from the city of Loja, southern Ecuador
Source: PLoS One. 2021 Oct 27;16(10):e0258454. doi: 10.1371/journal.pone.0258454 (PMC8550592; doi:10.1371/journal.pone.0258454)
Supplement: S1 Fig — (PDF) [file pone.0258454.s001.pdf]

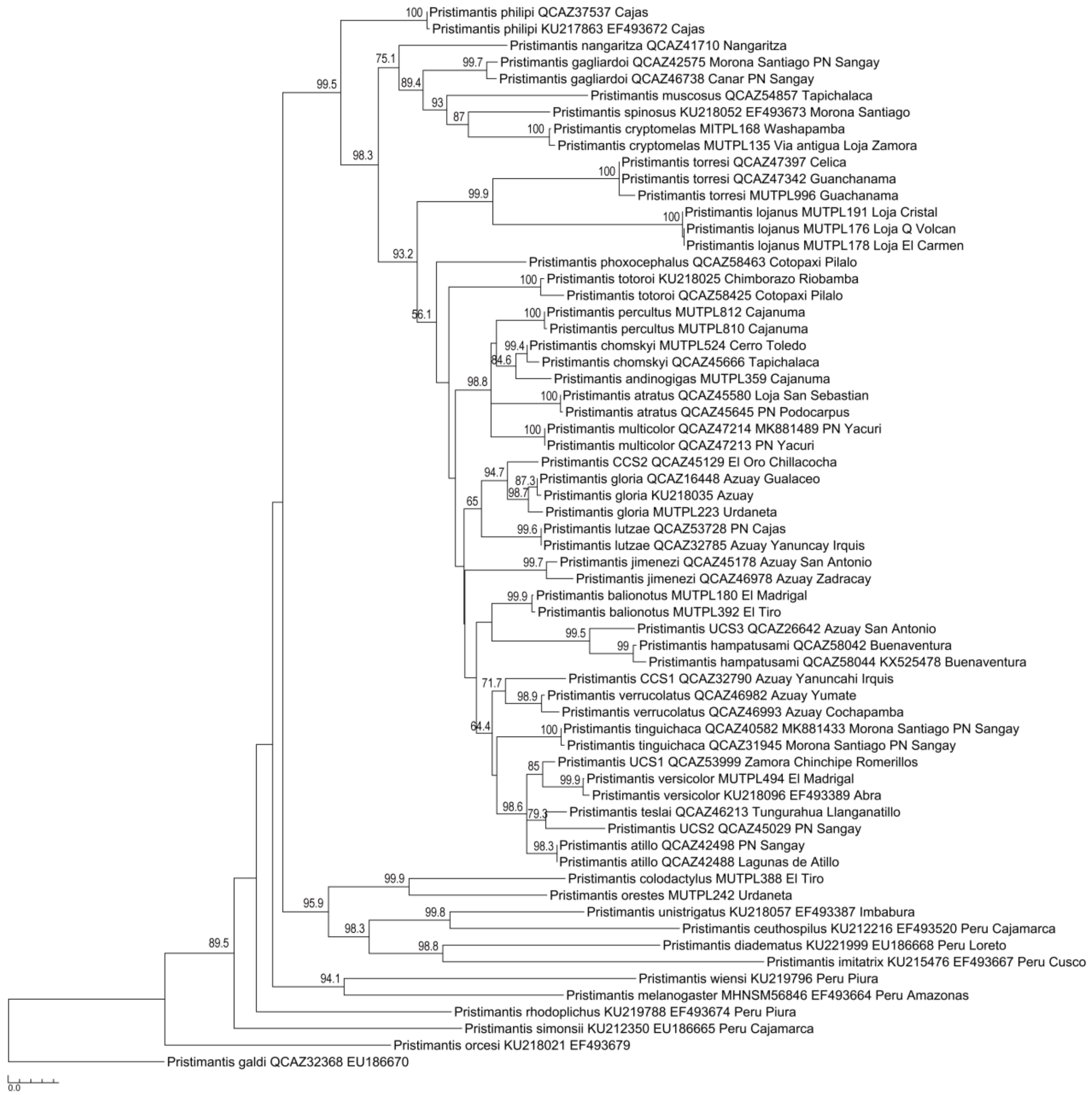

S1 Fig. Maximum likelihood phylogram including outgroup, based on 2339 base pairs of concatenated DNA from *12S*, *16S*, and *RAG-1* gene fragments.
