# Supplementary material for: Our unknown neighbor: A new species of rain frog of the genus Pristimantis (Amphibia: Anura: Strabomantidae) from the city of Loja, southern Ecuador
Source: PLoS One. 2021 Oct 27;16(10):e0258454. doi: 10.1371/journal.pone.0258454 (PMC8550592; doi:10.1371/journal.pone.0258454)
Supplement: S1 Table — (DOCX) [file pone.0258454.s003.docx]

**S1 Table.** **Voucher, GenBank accession numbers and locality for the specimens used in the phylogenetic analysis.**

| **Species** | **Voucher number** | **GenBank accession no.** | | | **Locality** |
| --- | --- | --- | --- | --- | --- |
|  |  | ***12S*** | ***16S*** | ***RAG1*** |  |
| *Pristimantis andinogigas* | MUTPL359 | MT764339 | MT756022 | MT810305 | Ecuador: Loja, Parque Nacional Podocarpus, Cajanuma |
| *Pristimantis atillo* | QCAZ42488 | - | MK881440 | MK881340 | Ecuador: Morona Santiago, Parque Nacional Sangay, Lagunas de Atillo |
| *Pristimantis atillo* | QCAZ42498 | - | MK881444 | MK881344 | Ecuador: Morona Santiago, Parque Nacional Sangay |
| *Pristimantis atratus* | QCAZ45580 | - | MK881471 | MK881364 | Ecuador: Loja, San Sebastián, Cajanuma |
| *Pristimantis atratus* | QCAZ45645 | - | MK881473 | MK881366 | Ecuador: Loja, Parque Nacional Podocarpus, Cajanuma |
| *Pristimantis balionotus* | MUTPL180 | MT778069 | MT756023 | MT810306 | Ecuador: Loja, Reserva Madrigal del Podocarpus |
| *Pristimantis balionotus* | MUTPL392 | MT778071 | MT756025 | MT810308 | Ecuador: Loja, Abra de Zamora |
| *Pristimantis chomskyi* | MUTPL524 | MZ678943 | MZ678934 | MZ700220 | Ecuador: Loja, Parque Nacional Podocarpus, Cerro Toledo |
| *Pristimantis chomskyi* | QCAZ45666 | - | MK881476 | MK881369 | Ecuador: Zamora Chinchipe, Reserva Tapichalaca |
| *Pristimantis cryptomelas* | MUTPL135 | MT778073 | MT756026 | MT810311 | Ecuador: Loja, Abra de Zamora |
| *Pristimantis cryptomelas* | MUTPL168 | MT778075 | MT756028 | MT810313 | Ecuador: Loja, Bosque Protector Washapamba |
| *Pristimantis gagliardoi* | QCAZ42575 | - | MK881456 | MK881355 | Ecuador: Morona Santiago, Parque Nacional Sangay, Ranger Station, Tinguichaca river |
| *Pristimantis gagliardoi* | QCAZ46738 | - | MK881480 | MK881372 | Ecuador: Cañar, Reserva Mazar |
| *Pristimantis gloria* | KU218035 | EF493348 | EF493348 | - | Ecuador: Azuay, 8.1 km W Morona Santiago border, Gualaceo-Limón road |
| *Pristimantis gloria* | MUTPL223 | MT778079 | MT756032 | MT810317 | Ecuador: Loja, 21 km E Urdaneta |
| *Pristimantis gloria* | QCAZ16448 | - | MK881402 | MK881316 | Ecuador: Azuay, Gualaceo-Macas road |
| *Pristimantis hampatusami* | QCAZ58042 | - | MK881504 | MK881387 | Ecuador: El Oro, Reserva Buenaventura |
| *Pristimantis hampatusami* | QCAZ58044 | - | KX525478 | KX525472 | Ecuador: El Oro, Reserva Buenaventura |
| *Pristimantis jimenezi* | QCAZ45178 | - | MK881468 | MK881362 | Ecuador: Azuay, San Antonio, Parque Nacional Cajas border |
| *Pristimantis jimenezi* | QCAZ46978 | - | MK881482 | MK881374 | Ecuador: Azuay, Molleturo, Zadracay river |
| *Pristimantis lojanus* | MUTPL176 | MZ678944 | MZ678935 | MZ700221 | Ecuador: Loja, Loja, Quebrada Volcan |
| *Pristimantis lojanus* | MUTPL178 | MZ678945 | MZ678936 | MZ700222 | Ecuador: Loja, Loja, Quebrada El Carmen |
| *Pristimantis lojanus* | MUTPL191 | MZ678946 | MZ678937 | MZ700223 | Ecuador: Loja, Cristal |
| *Pristimantis lutzae* | QCAZ32785 | - | MK881421 | MK881326 | Ecuador: Azuay, Bosque Protector Yanuncay-Irquis, Páramo de Quimsacocha |
| *Pristimantis lutzae* | QCAZ53728 | - | MK881495 | - | Ecuador: Azuay, Parque Nacional Cajas, El Capo, Laguna Toreadora |
| *Pristimantis multicolor* | QCAZ47213 | - | MK881488 | - | Ecuador: Loja, Parque Nacional Yacuri, Laguna Negra |
| *Pristimantis multicolor* | QCAZ47214 | - | MK881489 | - | Ecuador: Loja, Parque Nacional Yacuri, Laguna Negra |
| *Pristimantis muscosus* | QCAZ54857 | - | MK881501 | MK881386 | Ecuador: Zamora Chinchipe, Reserva Tapichalaca |
| *Pristimantis nangaritza* | QCAZ41710 | - | MK881436 | MK881336 | Ecuador: Zamora Chinchipe, Alto Nangaritza PF, Las Orquídeas, Tepuy forest |
| *Pristimantis percultus* | MUTPL810 | MT778088 | MT756034 | MT810325 | Ecuador: Loja, Parque Nacional Podocarpus, Cajanuma |
| *Pristimantis percultus* | MUTPL812 | MT778089 | MT756035 | MT810326 | Ecuador: Loja, Parque Nacional Podocarpus, Cajanuma |
| *Pristimantis philipi* | KU217863 | EF493672 | EF493672 | - | Ecuador: Azuay, 4 km W Laguna Toreadora, nearby Parque Nacional Cajas |
| *Pristimantis philipi* | QCAZ37537 | - | MK881426 | MK881331 | Ecuador: Azuay, Parque Nacional Cajas |
| *Pristimantis phoxocephalus* | QCAZ58463 | - | MK881507 | MK881390 | Ecuador: Cotopaxi, Pilaló surroundings |
| *Pristimantis spinosus* | KU218052 | EF493673 | EF493673 | - | Ecuador: Morona Santiago, 10.6 km W Plan de Milagro |
| *Pristimantis teslai* | QCAZ46213 | - | MK881478 | - | Ecuador: Tungurahua, Llanganatillo, Parque Nacional Llanganates border |
| *Pristimantis tinguichaca* | QCAZ31945 | - | MK881418 | MK881323 | Ecuador: Morona Santiago, Parque Nacional Sangay, San Vicente |
| *Pristimantis tinguichaca* | QCAZ40582 | - | MK881433 | MK881334 | Ecuador: Morona Santiago, Parque Nacional Sangay, Lagunas de Atillo |
| *Pristimantis torresi* | MUTPL996 | MZ678947 | MZ678938 | MZ700224 | Ecuador: Loja, Guachanamá, El Apretadero |
| *Pristimantis torresi* | QCAZ47342 | - | MK881490 | - | Ecuador: Loja, Guachanamá, military antennas |
| *Pristimantis torresi* | QCAZ47397 | - | MK881492 | MK881380 | Ecuador: Loja, Celica-Alamor road |
| *Pristimantis totoroi* | KU218025 | EF493349 | EF493349 | - | Ecuador: Chimborazo, 70 km W Riobamba via Pallatanga |
| *Pristimantis totoroi* | QCAZ58425 | - | MK881505 | MK881388 | Ecuador: Cotopaxi, Pilaló surroundings |
| *Pristimantis verrucolatus* | QCAZ46982 | - | MK881483 | MK881375 | Ecuador: Azuay, Yumate, Shoupshe |
| *Pristimantis verrucolatus* | QCAZ46993 | - | MK881485 | MK881377 | Ecuador: Azuay, Cochapamba |
| *Pristimantis versicolor* | KU218096 | EF493389 | EF493389 | EF493431 | Ecuador: Loja, Abra de Zamora (1.7 km E Zamora Chinchipe-Loja border) |
| *Pristimantis versicolor* | MUTPL494 | MT778095 | MT756038 | MT810332 | Ecuador: Loja, Reserva Madrigal del Podocarpus |
| *Pristimantis* sp. CCS1 | QCAZ32790 | - | MK881423 | MK881328 | Ecuador: Azuay, Bosque Protector Yanuncay-Irquis, Páramo de Quimsacocha |
| *Pristimantis* sp. CCS2 | QCAZ45129 | - | MK881462 | MK881358 | Ecuador: El Oro, Chillacocha |
| *Pristimantis* sp. UCS1 | QCAZ53999 | - | MK881496 | - | Ecuador: Zamora Chinchipe, Yacuambi, Romerillos |
| *Pristimantis* sp. UCS2 | QCAZ45029 | - | MK881461 | - | Ecuador: Morona Santiago, Parque Nacional Sangay, Etén, Rio Culebrillas |
| *Pristimantis* sp. UCS3 | QCAZ26642 | - | MK881409 | - | Ecuador: Azuay, San Antonio de Chaucha |
