# Supplementary material for: Our unknown neighbor: A new species of rain frog of the genus Pristimantis (Amphibia: Anura: Strabomantidae) from the city of Loja, southern Ecuador
Source: PLoS One. 2021 Oct 27;16(10):e0258454. doi: 10.1371/journal.pone.0258454 (PMC8550592; doi:10.1371/journal.pone.0258454)
Supplement: S1 Appendix — (DOCX) [file pone.0258454.s006.docx]

**S1 Appendix. Additional specimens examined.**

*Pristimantis andinogigas.* ECUADOR: LOJA PROVINCE, Parque Nacional Podocarpus - Cajanuma (MUTPL 359).

*Pristimantis atratus.* ECUADOR: LOJA PROVINCE, Abra de Zamora (MUTPL 210, 321–323); Loja, Huacapamba (MUTPL 382); ZAMORA CHINCHIPE PROVINCE, Reserva Tapichalaca (MUTPL 220, 291).

*Pristimantis balionotus.* ECUADOR: LOJA PROVINCE, Abra de Zamora (MUTPL 292, 297, 391, 392, 677); Reserva Madrigal del Podocarpus (MUTPL 487, 489–491).

*Pristimantis chomskyi.* ECUADOR: LOJA PROVINCE, Parque Nacional Podocarpus - Cerro Toledo (MUTPL 113–116, 121, 524–526, 816).

*Pristimantis cryptomelas.* ECUADOR: LOJA PROVINCE, Abra de Zamora (MUTPL 135, 470, 471); Bosque Protector Washapamba (MUTPL 167–171); Loja, Huacapamba (MUTPL 380, 381, 383, 385); Parque Nacional Podocarpus - Cajanuma (MUTPL 493).

*Pristimantis gloria.* ECUADOR: LOJA PROVINCE, Vía Urdaneta-Tutupali (MUTPL 222–238, 250); ZAMORA CHINCHIPE PROVINCE, Belen, Bosque Protector Shincata (MUTPL 801); MORONA SANTIAGO, Bosque Protector Jima (MUTPL 39–72).

*Pristimantis percultus.* ECUADOR: LOJA PROVINCE, Parque Nacional Podocarpus - Cajanuma (MUTPL 810–812).

*Pristimantis torresi.* ECUADOR: LOJA PROVINCE, Guachanamá, El Apretadero (MUTPL 996–998).

*Pristimantis versicolor.* ECUADOR: LOJA PROVINCE, Abra de Zamora (MUTPL 112, 293, 294, 313, 389, 390, 497); Loja, Huacapamba (MUTPL 806); Parque Nacional Podocarpus - Cajanuma (MUTPL 910); Ramos Urcu (MUTPL 719); Reserva Madrigal del Podocarpus (MUTPL 494); ZAMORA CHINCHIPE PROVINCE, Reserva Tapichalaca (MUTPL 738–740); Reserva Cerro Plateado (MUTPL 653).
